# *In vitro* gastrointestinal digestion simulation screening of novel ACEI peptides from broccoli: mechanism in high glucose-induced VSMCs dysfunction

**DOI:** 10.3389/fnut.2025.1528184

**Published:** 2025-01-27

**Authors:** Shuzhi Zhang, Jingjing Guo, Shikun Suo, Li Ju, Zhaoqiang Jiang, Pingshuan Dong, Yanli Wang, Yali Dang, Laijing Du

**Affiliations:** ^1^School of Phamacy, Hangzhou Medical College, Hangzhou, China; ^2^Luoyang Key Laboratory of Cardiovascular Science, The First Affiliated Hospital, and College of Clinical Medicine of Henan University of Science and Technology, Luoyang, China; ^3^College of Food Science and Engineering, Ningbo University, Ningbo, China; ^4^Henan Provincial Key Laboratory of Cardiovascular Disease Medicine, The First Affiliated Hospital, and College of Clinical Medicine of Henan University of Science and Technology, Luoyang, China

**Keywords:** gastrointestinal digestion simulation, ACEI peptides, broccoli, VSMCs, Ang II, ACE2

## Abstract

Many natural angiotensin-converting enzyme inhibitory (ACEI) peptides have been widely studied. However, their stability *in vivo* is poor in most cases. In this study, peptides were initially digested from broccoli *in vitro*, and absorption was simulated by Caco2 cells transport and then analyzed by Peptideomics and molecular docking. Subsequently, the mechanisms were verified using a high glucose-induced vascular smooth muscle cells (VSMCs) dysfunction model. Results showed that ACEI activity of broccoli crude peptide increased by 70.73 ± 1.42% after digestion. The enzymatic hydrolysates of crude broccoli peptides before and after digestion were detected by HPLC. The digested crude peptides were highly stable (with a stability level > 90%) in the intestine and possessed a strong absorptive potential. Five peptides with high stability and strong permeability were first identified, including HLEVR, LTEVR, LEHGF, HLVNK, and LLDGR, which exhibited high activity with IC_50_ values of 3.19 ± 0.23 mM, 17.07 ± 1.37 mM, 0.64 ± 0.02 mM, 0.06 ± 0.01 mM, and 2.81 ± 0.12 mM, respectively. When the VSMCs model was exposed to Ang II, the expressions of PCNA, MMP2, and Bcl2 were increased, while the expression of BAX was inhibited. When the VSMCs was exposed to high glucose (HG), the Ang II concentration significantly increased. This indicates that HG elevated Ang II levels. Finally, five peptides significantly attenuated Ang II-induced VSMCs proliferation and migration by down-regulating AT1R expression and inhibiting ERK and p38 MAPK phosphorylation. Notably, in exploring VSMCs dysfunction on a high glucose-induced model, ACEI peptides resulted in down-regulation of ACE and up-regulation of ACE2 expression. Therefore, it can be further referenced for the functional food against hypertension and cardiovascular diseases.

## 1 Introduction

ACE is a pivotal enzyme that has a regulatory effect on blood pressure in human renin-angiotensin system (RAS) (1). By inhibiting ACE activity, it can reduce the production of angiotensin II (Ang II), which binds to receptors on the vascular wall, causing vasoconstriction can be reduced (2). Under conditions of high glucose, increased ACE activity mainly promotes inflammation and oxidative stress through upregulation of the angiotensin II type 1 (AT1) receptor and accelerates the production of more Ang II in atherosclerosis (3). This affects the transforming growth factor-beta (TGF-β) signaling pathway, promoting fibrosis, vascular wall thickening, cell proliferation, and impacting vascular elasticity (4). Concurrent activation of the renin-angiotensin-aldosterone system (RAAS) leads to water and sodium retention, increasing blood volume and further elevating blood pressure. Accumulating evidence suggests that, the proliferation and migration of VSMCs are regulated by angiotensin II (Ang II), which is produced by ACE (5–9). Common ACEIs include captopril, lynopril, enalapril, etc., which are used in the clinical management of hypertension to lower blood pressure in hypertensive patients (10). In addition to synthetic ACEI drugs, natural ACEI bioactive peptides have also received widespread attention.

Broccoli is a globally significant crop that is recognized for its health benefits due to its various bioactive compounds and high protein content (11). Bioactive peptides from broccoli protein have shown potential in alleviating cardiovascular and metabolic diseases (12). Chen et al. screened broccoli as the vegetable with the highest angiotensin I converting enzyme (ACE) inhibitory activity from 50 kinds of vegetables. The novel ACE inhibitory peptide KSVLLKF in broccoli has potential antihypertensive applications in functional foods/drugs (13). Li et al. extracted ACEI peptide from broccoli protein hydrolysate, and through the experimental method of peptiomics and 3D-QSAR model combined with experimental verification. The results showed that two novel peptides, FVLPLR and LPWYR, could reduce blood pressure (14).

Furthermore, in our previous study, ACEI peptides (LPGVLPVA and YLYSPAY) from broccoli significantly reduced the blood pressure of spontaneously hypertensive rats and the glucose level in a mouse model (15). This finding implies that ACEI peptides from broccoli have the potential to improve hypertension and hyperglycemia.

Peptides are susceptible to degradation and inactivation by gastrointestinal digestive enzymes (16). Generally, long peptides or those obtained through enzymatic hydrolysis using conventional methods are easily broken down and lose their activity in the gastrointestinal tract, leading to ineffective absorption in the intestines (17). The peptides derived from simulated gastrointestinal tract digestion exhibit remarkable stability (16). The Caco-2 cell transport model serves as a valuable tool for assessing the stability and bioactivity of peptides in the intestinal environment (18). Fan et al. identified the stability and antihypertensive activity of Spent Hen-Derived ACE-Inhibitory Peptides IWHHT, IWH, and IWWHHT, IWH, and IW using a Caco-2 cell model (19). Zhao et al. employed LSGYGP isolated from tilapia skin gelatin hydrolysate to determine the antihypertensive effect of LSGYGP through the Caco-2 cell model *in vivo* (20). These studies indicate that Caco2 cell transport model is an effective means to investigate the absorption function of bioactive peptides.

In the context of high glucose, various strategies have been employes to mitigate the effects of ACE and Ang II. Angiotensin II type-2 receptor (AT2R) signaling typically promotes vasodilation or growth inhibition, alleviating inflammation (21, 22). ACE protects human islets from significant glucose toxicity, and strategies aimed at lowing blood sugar levels have proven effective (23). But each approach carries potential side effects, such as coughing, decreased kidney function, elevated blood potassium, gastrointestinal irritation, and increased cardiovascular risk. Therefore, the development of natural ACE inhibitors with low side effects is an urgent need for cardiovascular therapy.

Peptide research has witnessed notable progress, yet existing studies harbor several significant gaps. The simplicity of past protein extraction methods likely limited the variety and quality of peptides for analysis. Additionally, the structural characteristics of identified novel peptide sequences have not been thoroughly investigated. Moreover, while previous research has explored the general effects of broccoli and its peptides, there is a dearth of specific studies on how peptides target Ang II under high—glucose conditions, including the underlying mechanisms and optimal treatment parameters.

In this study, the absorption properties of peptides were completely simulated using *in vitro* gastrointestinal simulation and the Caco-2 cell transport model. By combining peptiomics with molecular docking, the ACEI peptides with high absorption potential and antihypertensive activity were screened. The Ang II model was employed to investigate the role of ACEI peptides in cellular signaling pathways and to observe their effects on the proliferation, migration, and phenotypic transformation of VSMCs. Concurrently, the high glucose model was utilized to evaluate the alterations in peptide levels concerning Ang II within the cells and to examine the impact of ACEI on VSMC proliferation, migration, and apoptosis under high glucose conditions. These studies provided theoretical references regarding the changes of VSMC cells in the presence of AngII and HG, offering new insights into cardiovascular protection for diabetic patients.

## 2 Materials and methods

### 2.1 Materials and reagents

Gastric protease and pancreatic protease were purchased from Dalian Meilun Biotechnology Co., Ltd. (Dalian, China). Angiotensin converting enzyme (ACE) and N-hippuryl-l-histidyl-l-leucine hydrate (HHL) were obtained from Sigma–Aldrich (Shanghai) Trading Co., Ltd. (Shanghai, China). The stems and leaves of broccoli (*B. oleracea var. italica*) were purchased from Zhejiang Tianlai Biotechnology Co., Ltd. (Zhejiang, China). Fetal bovine serum (FBS) was purchased from Gibco Co., Ltd. (Grand Island, USA). Dulbecco's modified Eagle's medium (DMEM), phosphate-buffered saline (PBS), and Cell Counting Kit-8 (CCK-8) were purchased from Solarbio Science & Technology Co., Ltd. (Beijing, China). Proliferating cell nuclear antigen (PCNA) mouse monoclonal antibody, Matrix Metallopeptidase 2 (MMP2) monoclonal antibody, β-actin, ERK1/2 rabbit polyclonal antibody, p38 MAPK polyclonal antibody, phospho-p38 MAPK (Thr180/Tyr182), polyclonal antibody phospho-ERK1/2 (Thr202/Tyr204) Ribbit polyclonal antibody, B-cell lymphoma-2 (Bcl2) monoclonal antibody, HRP-conjugated AffiniPure goat anti-rabbit IgG (H+L), and HRP-conjugated AffiniPure goat anti-IgM Fluorescein isothiocyanate (FITC) and the BeyoClickTM EdU Cell Proliferation Kit with Alexa Fluor 488 were purchased from Beyotime Biotechnology Co., Ltd. (Shanghai, China). Collagenase-II was obtained from Biosharp Co., Ltd. (Guangdong, China).

### 2.2 Preparation of broccoli peptides

#### 2.2.1 Preparation of broccoli protein

Fresh broccoli stems and leaves were juiced, and the juice was centrifuged at 5,000 rpm for 10 min at 4°C. The supernatant was heated for 15 min at 95°C and cooled to room temperature. The precipitate was collected by centrifugation at 8,000 rpm for 10 min at 4°C and freeze-dried to obtain the broccoli protein sample.

#### 2.2.2 *In vitro* digestion simulation of broccoli protein

*In vitro* digestion simulation was performed based on the method of Hao et al. (24). Broccoli protein suspension (5%, w/v) was hydrolyzed with papain (1%, E/S ratio) for 6 h (pH 7.0, 55°C). After hydrolysis, inactivated at 100°C for 10 min and cooled to 25°C. Based on the method of Hao et al. (24), the simulated digestion of broccoli papain hydrolysate was conducted. In short, 0.1 g pepsin (pH = 2.0) was mixed with the hydrolysate and placed at 37°C for 2 h. Then 0.2 g trypsin (pH = 8.0) was added and incubated at 37°C for 2 h. After enzymatic hydrolysis, the enzyme was inactivated at 95°C for 10 min, cooled to 25°C, and the supernatant was collected. Subsequently, freeze-dried broccoli crude peptide was applied to the determination of ACEI activity.

The crude protein hydrolysis of broccoli was analyzed by high performance liquid chromatography (Agilent 1260, California, USA). The Column was XBridge Peptides BEH C18 Column 130A, 4.6 × 250 mm, 5 μm (Waters, Massachusetts, USA). Column temperature: 37°C. Flow rate: 1 mL/min. Sample size: 100 μL. Mobile phase: 0.1% trifluoroacetic acid aqueous solution (phase A) and pure acetonitrile (phase B). Detection wavelengths: 214 and 280 nm. Elution gradient: 0–5 min, 100%A/0%B; 5–25 min, 60%A/40%B; 25–26 min, 20%A/80%B; 26–31 min, 20%A/80%B; 31–32 min, 100%A/0%B; 32–37 min, 100%A/0%B.

#### 2.2.3 ACEI activity of broccoli peptides before and after digestion

Eighty microlitres of HHL solution (5 mM) and 30 μL of sample mixture were incubated at 37°C for 5 min. Then, 40 μL of ACE solution (0.025 U/mL) was added to the mixture, followed by incubation at 37°C for 60 min. The reaction was terminated by adding 120 μL of 1 M HCl. After the addition of 700 μL of pure water, the mixture was filtered through a 0.22 μm membrane (25). The experiment included four groups: a control group (pure water), a negative control group (blank digestion solution), a positive control group (protein mixture), and a sample group (protein digestion solution). In the control group, HCl was added immediately after ACE.

Chromatograms were recorded via an Agilent instrument (Agilent, California, USA). Column: CAPCELL CORE AQ C18, 4.6 × 150 mm, 5 μm (Shiseido, Tokyo, Japan). Column temperature: 30°C. Flow rate: 1 mL/min. The injection volume was 100 μL. Mobile phase: 0.1% trifluoroacetic acid aqueous solution (A) and pure acetonitrile (B). Detection wavelength: 228 nm. Elution gradient: 0–10 min, 80% A/20% B. The calculation method was as follows:


$$\begin{array}{l} \text{Inhibitionrate}\left(\text{\%}\right)={\left[\text{PA}\right]}_{\text{c}}-{\left[\text{PA}\right]}_{\text{s}}/{\left[\text{PA}\right]}_{\text{c}}-{\left[\text{PA}\right]}_{\text{b}}\times 100\text{\%} \end{array}$$

where [PA]_c_, [PA]_s_, and [PA]_b_ represented the peak area of HA at 228 nm of the control group, experimental group, and blank group, respectively.

#### 2.2.4 Absorption simulation of broccoli peptides

Caco-2 cells were obtained from the Cell Bank of the Chinese Academy of Sciences. The cells were cultured in Dulbecco's modified Eagle's medium (DMEM) supplemented with 20% FBS and 1% penicillin/streptomycin (P/S) at 37°C and 5% CO_2_ in a humidified incubator.

Caco-2 cells were seeded in each chamber of a 12-well plate, which was equipped with cell culture inserts (26). The transepithelial electrical resistance (TEER) across the monolayer was measured every 2 days via a cell resistance meter (RE1600, Beijing Jegon Instrumentation Co., Ltd.). The integrity of the cell monolayer was assessed with fluorescein isothiocyanate (FITC). TEER values > 350 Ω/cm^2^ were considered to indicate successful construction and validation of the Caco-2 cell transport model. The FITC permeability was < 5 × 10^−7^ cm^2^/s, indicating the integrity of the cell monolayer.

Broccoli protease hydrolysate was added to the apical (AP) side. After 120 min, the media from both the AP and basolateral (BL) sides were collected for subsequent analysis. The wells without cells or samples served as the control group, whereas the wells without samples served as the blank group.

### 2.3 Screening and synthesis of broccoli ACEI peptides

#### 2.3.1 Identification of broccoli peptides by peptideomics

Mass analysis of the peptides was conducted according to the methods of Li et al. (27). The media were analyzed by mass spectrometry. The identified peptides were ranked according to their intensity. The experiment was divided into sample group and blank group, in which blank group was blank medium. After removing sequences that matched those in the blank group, the top four peptide sequences whose intensities were higher than those in the blank group by at least four orders of magnitude were selected, which were then matched with all known broccoli proteins via Python.

#### 2.3.2 Screening and synthesis of broccoli peptides

The peptides used for screening and synthesis were modified from the methods of Chen et al. (28). The three-dimensional structures of the peptide segments were generated via the RDkit database within Python 3.9.2, followed by energy minimization via the MMFF94s force field. The optimized structures are converted into PDB format. The X-ray crystallographic structure of human ACE (PDB ID: 1O8A, resolution = 2.0 Å) was subsequently retrieved from the protein database PDB and saved as a PDB file. All water molecules and heteroatoms of ACE were removed, and hydrogen atoms were added. After the peptide segment was imported, hydrogenation was performed, and the rotatable bonds were appropriately set. The center-*x* = 43.817, center-*y* = 38.308, center-*z* = 46.652, and redium = 15 Å were designated as the docking sites, and semiflexible docking was executed via AutoDock Vina 1.2.5. The docking outcomes were subsequently visualized in three dimensions via PyMOL (Version 2.6.0; Schrödinger, LLC) and in two dimensions via Discovery Studio (Version 20.1; Dassault Systèmes BIOVIA).

Finally, five peptides with potential ACEIs were synthesized by Shanghai Apeptide Co., Ltd. (Shanghai, China), and the purities were maintained above 95%.

### 2.4 Effects of five screened ACEI peptides from broccoli on VSMCs

#### 2.4.1 Culture of VSMCs

Sprague–Dawley rats (5–8 weeks old) were purchased from HFK Bioscience Company (Beijing, China) and were anesthetized with tribromoethanol (300 mg/kg). All procedures were performed in accordance with the guidelines set by the Institutional Animal Care and Use Committee of the First Affiliated Hospital of Henan University of Science and Technology. The experimental design is compliant with ethical principles, protects the safety of experimental personnel, and simultaneously maintains the welfare of the animals (2020-12-K0080).

Thoracic aortas from male Sprague–Dawley rats (250–300 g) were dispersed in HBSS solution, which contained 1 mg/mL collagenase II (146 U/mg, Worthington Biochemical Corp., Freehold, NJ) and 0.5 mg/mL elastase I (32 U/mg, Worthington Biochemical Corp., Freehold, NJ). The mixture was digested for 90 min to obtain the appropriate VSMCs. The obtained VSMCs were subsequently cultured in DMEM supplemented with 10% FBS, 2 mmol/L glutamine, and 1 g/L D-glucose at 37°C in a humidified atmosphere of 5% CO_2_. The purity of the VSMCs was evaluated by the smooth muscle cell-specific marker SM22α (>97% for the stained cells). All the VSMCs used for the experiments were between the 3rd and 8th passages.

#### 2.4.2 CCK-8 assay

The cells (4 × 10^3^ cells/well) were seeded into 96-well plates and cultured overnight. The cells were subjected to serum deprivation for 24 h. Then, the culture mixture was removed, and 100 μL of free FBS culture mixture was added to the above culture mixture. Ten microliters of CCK-8 solution was added to each well. The plates were read with a BioTek Epoch 2 Microplate Spectrophotometer (Agilent Technologies, California, USA). The experiment was organized into seven groups, which included a normal group, a high glucose group, and peptide segments: HLEVR, LTEVR, LEHGF, HLVNK, and LLDGR. These segments were tested at concentrations of 0.0313, 0.0625, 0.125, 0.25, and 0.5 mg/mL.

#### 2.4.3 EdU detection of VSMCs

VSMCs proliferation was evaluated with 5-ethynyl-2'-deoxyuridine (EdU) incorporation assay. The cells (2.5 × 10^4^ cells/400 μL/well) were seeded into 12-well plates, cultured overnight, and subjected to serum deprivation for 24 h. Then, 400 μL of culture was removed, and 200 μL of 2X EdU solution was added to each well. The mixture was incubated for 2 h at room temperature (RT). The cells were fixed with 4% paraformaldehyde for 15 min and then permeabilized with 0.3% Triton X-100 in PBS for 15 min. The cells were subsequently washed in PBS with 3% BSA (5 min, RT, three times). Two hundred microliters of 1X click reaction mixture was added to each well (30 min, RT, dark). The cells were rinsed with a solution of PBS containing 3% BSA (5 min, RT, three times). The nuclei of the cells were incubated with 1X Hoechst 33342 (10 min, RT, dark). The cells were washed again in PBS with 3% BSA (5 min, RT, three times). Finally, the cells were photographed with a fluorescence microscope (Olympus Corporation, Tokyo, Japan) at 50 × magnification.

#### 2.4.4 Migration assay of VSMCs

VSMCs were seeded and grown to 80% confluency in 6-well plates (1.5 × 10^5^ cells/well), and then, the cells were subjected to serum deprivation for 24 h. After serum starvation, standard wounds (< 3 mm) were formed via a 200 μL sterile plastic pipette tip (the time point was set as 0 h). The medium was replaced with fresh medium three times, and the cells were cultured with 2% FBS in DMEM. Images of the wounds were taken at 0 and 24 h.

#### 2.4.5 Apoptosis analysis of VSMCs

Cell apoptosis was detected with a FITC-Annexin V apoptosis detection kit (Proteintech, Wuhan, China). Briefly, the cells were washed with cold PBS three times and were digested with 0.25% trypsin. The cells were washed with cold PBS three times, and then 5 μL of FITC/Annexin V and 5 μL of propidium iodide were added to each tube (15 min, RT, dark). The cells were analyzed via Attune CytPix flow cytometry (Invitrogen, California, USA).

The cells (2 × 10^4^ cells per well) were first seeded into 24-well plates overnight. The supernatants were collected to measure the expression levels of Ang II via angiotensin II ELISA kits (ER1637, FineTest, Wuhan, China) according to the manufacturer's protocol. The absorbance of each well at 450 nm was determined via a multimode plate reader (BioTek Epoch2, Santa Clara, US).

#### 2.4.6 Western blot

Protein was extracted with RIPA buffer. Equal amounts of protein were separated via 8–10% SDS–PAGE (ACE, Changzhou, China) and then transferred to 0.22 μm PVDF membranes (Millipore, Bedford, MA, USA). The membranes were blocked with 5% skim milk and incubated overnight with the appropriate primary antibodies. HRP-conjugated secondary antibodies were then applied for binding and visualization through Western blot luminol reagent. Densitometry quantification was performed with ImageJ-V1.8.0.

### 2.5 Statistical analysis

All the statistical analyses were performed via GraphPad 9.5 software (GraphPad Software, Inc., USA). The data were analyzed by ANOVA, and an unpaired test was used for multiple comparisons. *P*-values < 0.05 were considered statistically significant.

## 3 Results and discussion

### 3.1 The content and ACEI activity of broccoli crude peptides

In this study, the protein content of broccoli was 61.39 ± 2.43 g/100 g. After *in vitro* digestion, the digestibility of broccoli protein was 44.23 ± 5.21%. This result indicated that broccoli protein could not be completely hydrolyzed to amino acids by a single gastric protease or pancreatic protease. The results of ACEI activity before and after digestion of broccoli protein are shown in Table 1. Compared with the protein before digestion, the ACEI activity of the protein after digestion was significantly increased by 70.73 ± 1.42%.

**Table 1:** ACEI activity before and after digestion of broccoli protein.

**Sample**	**ACE inhibitory activity (%)**
Control	0.89 ± 0.12^a^
Before digestion	1.38 ± 0.05^b^
After digestion	72.11 ± 1.74^c^

There were significant differences among different letters (*P* < 0.05).

Studies have shown that the active peptides in broccoli have a variety of biological functions, such as antioxidant, anti-inflammatory, and antihypertensive effects (14, 27, 29). For example, some studies extracted broccoli peptides by enzymatic hydrolysis and found that they can effectively inhibit ACE activity, showing good potential for blood pressure reduction (14, 30, 31). In this study, the same results were shown.

### 3.2 Absorption stability of broccoli peptides by Caco-2

Caco-2 transport modeling was performed to verify the ability of broccoli to absorb crude protein in the intestine. The final transepithelial electrical resistance (TEER) value of the chamber was 468.09 ± 37.54 Ω/cm^2^, and it remained unchanged for several consecutive days. The apparent permeability coefficient (Papp) of lucifer yellow was 2.87 ± 0.53 × 10^−^7 cm^2^/s, which was < 5.0 × 10^−^7 cm^2^/s. The results revealed that the TEER values of the cell culture inserts were > 350 Ω/cm^2^, indicating that the integrity of the cell monolayer was maintained. Peptidomics analysis revealed that broccoli peptides could cross the monolayer of Caco-2 cells and enter the BL side (Supplementary Table S1). This suggested that broccoli peptides were able to be absorbed by the intestinal tract, thus exerting their biological activity.

### 3.3 Identification of ACEI peptides in broccoli after simulated digestion and absorption

Through comprehensive analysis of protein composition and content, proteomics and peptidyomics improve the accuracy of the extraction and purification process and thus provide a theoretical basis for the development of functional components and promote the development of new health products (32, 33). The obtained peptide sequences and protein sequences of broccoli are listed in Supplementary Tables S1, S2. Owing to the weaker cutting action of pepsin and the stronger cutting action of pancreatin, the C-terminal amino acids of the obtained peptide sequences were almost all arginine (Arg) and lysine (Lys) (25). Arg and Lys have positive charge and can bind with carboxyl group of ACE active site, enhance the affinity of antihypertensive peptide and ACE, and improve the inhibitory effect (34). From the point of view of charge interaction, the active center of ACE has negative charge, while the side chain amino group of Arg and Lys is positive. When the ratio of Arg to Lys in ACE inhibitor peptide was appropriate, it could form strong electrostatic interaction with ACE active center, thus enhancing the binding force between ACE inhibitor peptide and ACE. The spatial structure and charge distribution of the antihypertensive peptide were changed, and the binding ability and inhibitory activity of the peptide with ACE were affected (35, 36). Other studies have shown that a high Arg:Lys ratio can lower the systolic blood pressure of hypertensive rats (26, 37, 38).

Furthermore, the high-frequency peptide sequences almost all contain leucine (Leu), which is influenced by the sequence of the broccoli protein itself. Some peptide sequences cannot be matched to known broccoli protein sequences (39). Different broccoli proteins were enzymatically cleaved into oligopeptides after digestion with pepsin and pancreatin. Among them, A0A249RRZ5_BRAOT and 6KEK2_BOT were rich in bioactive peptides. These two protein sequences were selected for the identification of subsequent peptides. The identification results of broccoli peptides by mass spectrometry are presented in Supplementary Table S3.

### 3.4 Screening of ACEI peptides by molecular docking and determination of ACEI activity and digestive stability

The prediction of the peptides with ACEI activity was shown in Table 2. The results were that the binding energies are −9.7 kcal/mol for HLEVR, −9.3 kcal/mol for LTEVR, −8.9 kcal/mol for HLVNK, −9.8 kcal/mol for LEHGF, and −8.9 kcal/mol for LLDGR. It indicated that these five peptides potentially exhibited ACEI activity. Lower ACEI energy corresponds to an increase in ACEI activity (40).

**Table 2:** ACE binding energy of broccoli peptide.

**Number**	**Peptides**	**–Energy (kcal/mol)**
1	HLEVR	9.7
2	LTEVR	9.3
3	LEHGF	9.8
4	HLVNK	8.9
5	LLDGR	8.9

**Figure 1:**
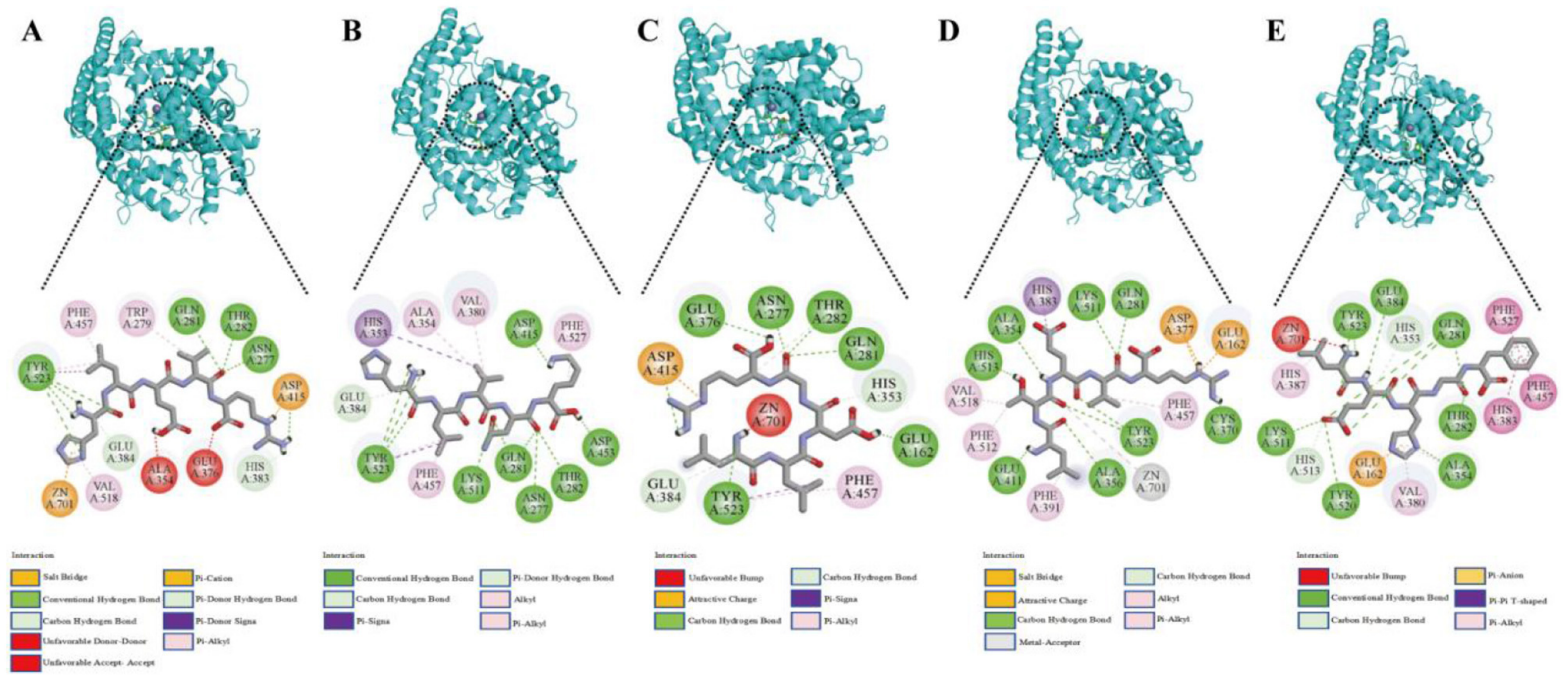
The expected interactions between the peptides and the ACE molecule. **(A)** HLEVR, **(B)** HLVNK, **(C)** LLDGR, **(D)** LTEVR, **(E)** LEHGF.

To investigate the molecular interactions between the ACEI peptides from broccoli and ACE, a visual analysis of the molecular docking results was conducted (Figure 1). The interaction between broccoli peptides and ACE was primarily mediated by hydrogen bonding interactions, hydrophobic interactions, and electrostatic interactions. Among them, hydrogen bonds play a key role in the complex formation of the five peptides and the amino acid residues of ACE. Specifically, 11, 12, 10, 10, and 8 hydrogen bonds were generated between ACE and the peptides (HLVNK, LEHGF, LLDGR, HLEVR, and LTEVR). Amino acid residues such as GLU384, TYR523, ASP415, LYS511, GLN281, ASN277, THR282, and ASP453 are located in the active site of ACE via hydrogen bonds. In addition, HLEVR, LTEVR, LLDGR, and HLVNK interacted with Zn^2+^ in ACE.

Through molecular docking, stable complexes were formed between HLEVR, LTEVR, LEHGF, HLVNK, LLDGR, and the receptor with potential ACEI activities. However, the inhibitory activities of these five peptides require further validation *in vitro*. The ACEI activities of the peptides are presented in Table 3. The ACEI activities of HLEVR, LTEVR, LEHGF, HLVNK, and LLDGR were 3.19 ± 0.23 mM, 17.07 ± 1.37 mM, 0.64 ± 0.02 mM, 0.06 ± 0.01 mM, and 2.81 ± 0.12 mM, respectively.

**Table 3:** ACEI activity and digestion stability of peptides.

**Number**	**Peptides**	**IC_50_ (mM)**	**Digestion stability (%)**
1	HLEVR	3.19 ± 0.23	94.27 ± 2.64
2	LTEVR	17.07 ± 1.37	98.82 ± 3.92
3	LEHGF	0.64 ± 0.02	97.85 ± 6.31
4	HLVNK	0.06 ± 0.01	95.78 ± 3.73
5	LLDGR	2.81 ± 0.12	98.82 ± 3.92

Peptides are converted to amino acids by digestive enzymes, thus diminishing their biological efficacy (41). Consequently, the stability of these peptides under digestion conditions was evaluated *in vitro* (Table 3). The digestion stabilities of HLEVR, LTEVR, LEHGF, HLVNK, and LLDGR all exceeded 90%, corresponding to 94.27 ± 2.64%, 98.82 ± 3.92%, 97.85 ± 6.31%, 95.78 ± 3.73%, and 98.82 ± 3.92%, respectively. These findings suggested that all five peptides displayed ACEI activity and digestion resistance.

### 3.5 ACEI peptides inhibited Ang II levels in VSMCs cultured in HG

**Figure 2:**
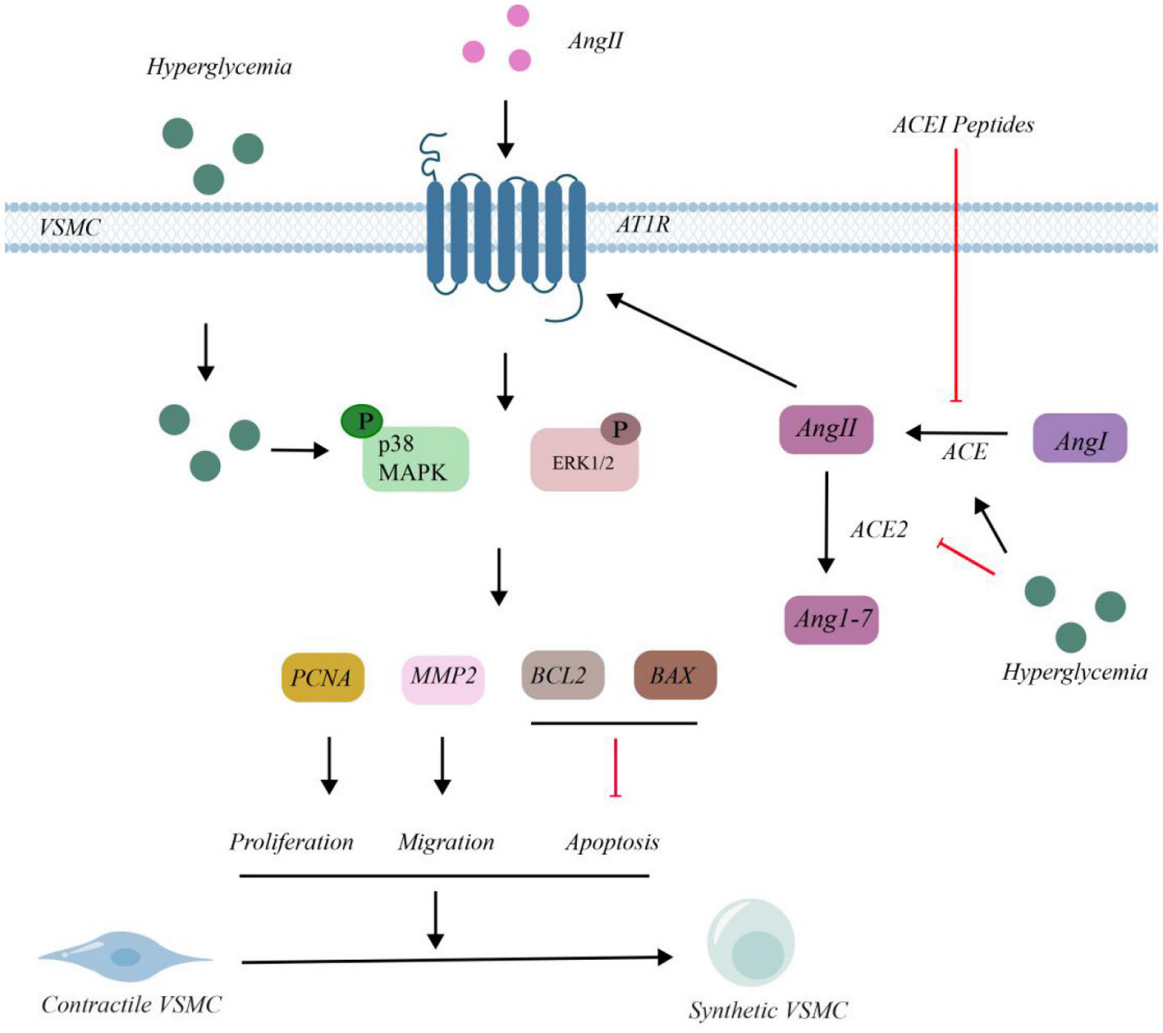
Molecular mechanism of VSMCs and intervention of ACEI peptide in HG environment.

The molecular mechanism of VSMCs and the intervention effect of ACEI peptide in high glucose environment are shown in the Figure 2. In VSMCs, under HG conditions, glucose enters the cells, leading to the phosphorylation of p38 and ERK1/2. Ang I is converted into Ang II under the action of ACE. Extracellular Ang II can also enter VSMCs through angiotensin II type 1 receptor (AT1R), which results in the phosphorylation of p38 and ERK1/2.

Under HG circumstances, Ang II was converted into Ang 1 - 7 by ACE2. The phosphorylation of p38 and ERK1/2 had upregulated the expressions of proliferating cell nuclear antigen (PCNA), matrix metalloproteinase 2 (MMP2), and B - cell lymphoma 2 (BCL2), while it had downregulated the expression of BCL2 - associated X protein (BAX). This series of changes ultimately led to the proliferation of VSMCs. HG treatment enhanced ACE activity and decreased ACE2 levels, leading to the accumulation of Ang II. After the intervention of ACEI peptides, the concentration of ACE decreased and the activity of ACE2 increased, thus reducing the accumulation of Ang II. As a consequence, the phosphorylation of p38 and ERK1/2 was inhibited, suppressing the expression of PCNA, MMP2, and BCL2, and increasing the expression of BAX. This indicated that these peptides could inhibit the proliferation and migration of VSMCs, thereby alleviating related symptoms.

#### 3.5.1 Effects of Ang II treatment on the expression of related proteins in VSMCs

**Figure 3:**
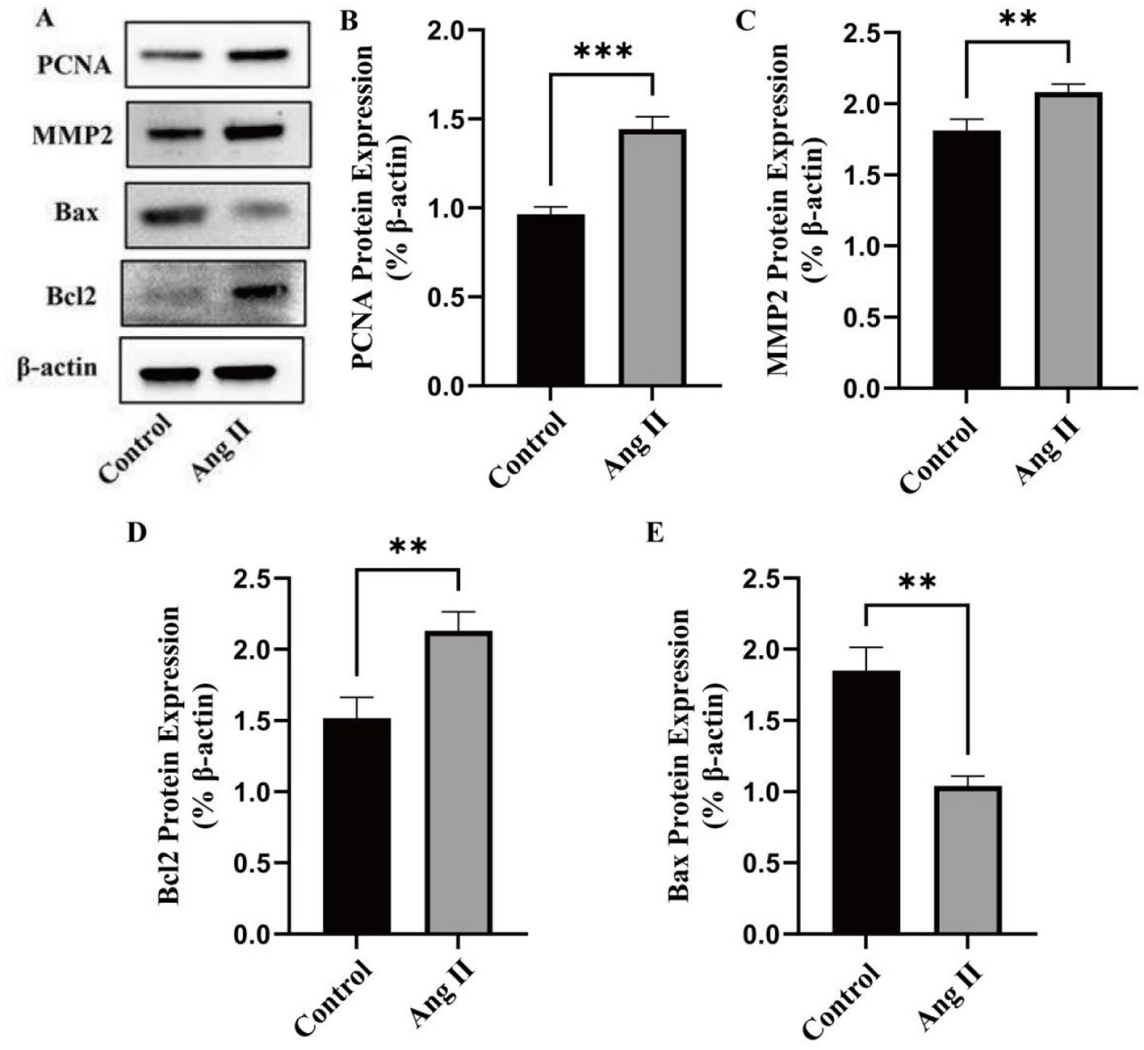
Effects of Ang II treatment on the expression of related proteins in VSMCs. **(A)** The expression of marker of proliferation (PCNA), migration (MMP2), and apoptosis (Bcl2 and Bax) in VSMCs induced by Ang II was evaluated by western blot. **(B)** Quantitative analysis of PCNA protein expression. **(C)** Quantitative analysis of MMP2 protein expression. **(D)** Quantitative analysis of Bcl2 protein expression. **(E)** Quantitative analysis of Bax protein expression. *P < 0.05; **P < 0.01; ***P < 0.001; ****P < 0.0001.

In the case of HG, the effect of Ang II on VSMCs is shown (Figure 3). Figures 3A–E illustrated the effects of Ang II treatment on the expression of related proteins in VSMCs under high-glucose (HG) conditions. The expression of PCNA, MMP2, and BCL2 was upregulated by 49.56 ± 0.01% (*P* < 0.001), 14.97 ± 0.044% (*P* < 0.01), and 40.26 ± 0.051% (*P* < 0.01), respectively, while the expression of Bax was downregulated by 43.78 ± 0.014% (*P* < 0.01). Ang II significantly increased the expression of PCNA and MMP2, indicating enhanced cell proliferation and matrix remodeling (5). Additionally, Ang II upregulated the pro-apoptotic protein Bax and downregulated the anti-apoptotic protein Bcl2, suggesting that Ang II treatment promotes cell apoptosis (42).

#### 3.5.2 Changes of Ang II level in VSMCs and culture medium under HG condition

**Figure 4:**
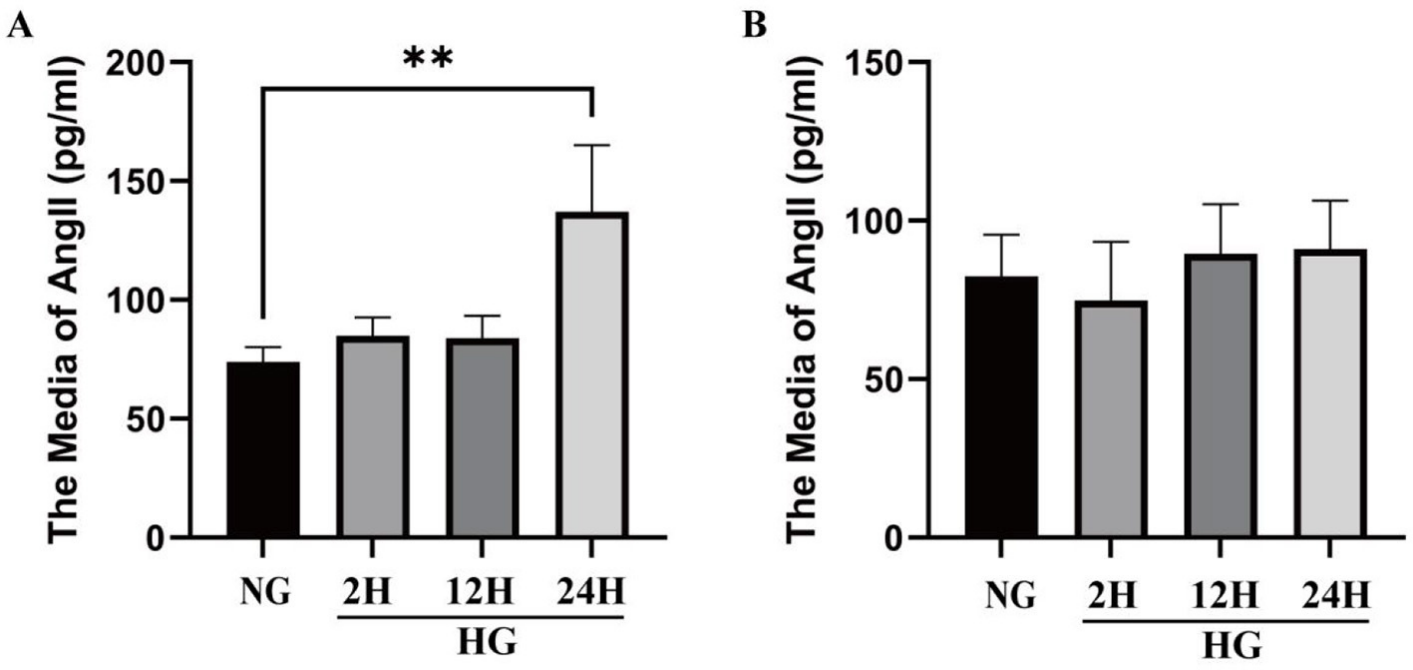
Changes of Ang II level in VSMCs and culture medium under HG condition. **(A)** The level of Ang II in VSMCs cultured in high glucose in different times in cell lysate. **(B)** The level of Ang II in VSMCs cultured in high glucose in different times in culture media. *P < 0.05; **P < 0.01; ***P < 0.001; ****P < 0.0001.

The changes of Ang II level in VSMCs and medium under HG condition are shown in Figure 4. Under HG conditions, Ang II protein levels were significantly higher than under normal glucose (NG) conditions, with a particularly marked increase at 24 h (Figure 4A). Compared with the NG group, the protein levels of Ang II in VSMCs increased by 14.92 ± 10.51%, 13.56 ± 12.63%, and 63.35 ± 37.86% (*p* < 0.01) respectively after exposure to HG conditions for 2, 12, and 24 h. The results indicate that high glucose induces elevated Ang II expression. And the media levels of Ang II increased by −9.26 ± 22.43%, 19.75 ± 20.94%, 1.61 ± 17.11% after exposure to HG for 2, 12, and 24 h. Figure 4B reveals that the Ang II level did not change significantly at different time points under HG conditions.

#### 3.5.3 Effects of ACEI peptides on Ang II level in HG environment

**Figure 5:**
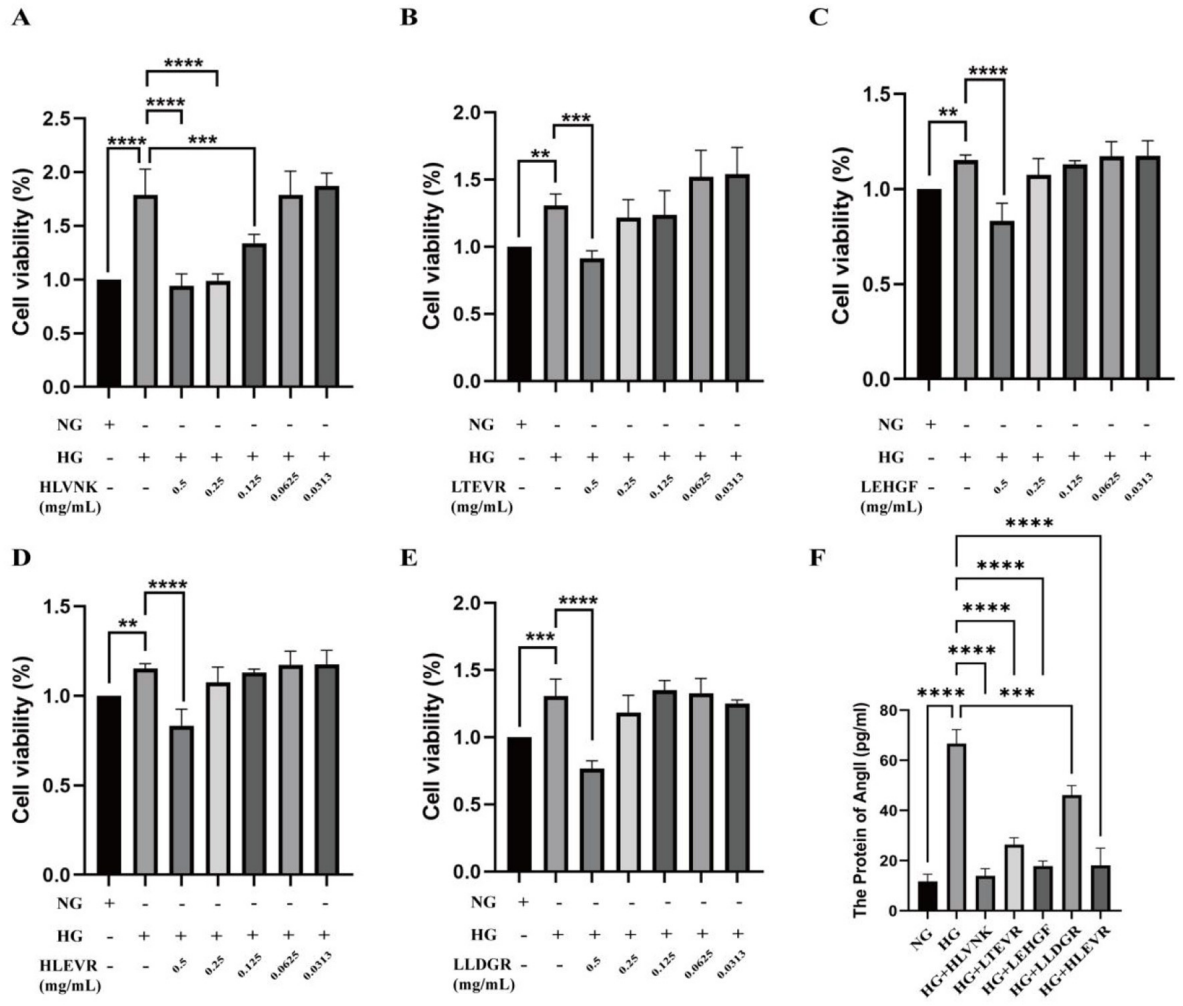
Effects of ACEI peptide on cell viability and Ang II level in HG environment. **(A)** Cell viability of HVLNK in HG environment. **(B)** Cell viability of LTEVR in HG environment. **(C)** Cell viability of LEHGF in HG environment. **(D)** Cell viability of HLEVR in HG environment. **(E)** Cell viability of LLDGR in HG environment. **(F)** Effect of ACEI peptide on Ang II protein expression in HG environment. *P < 0.05; **P < 0.01; ***P < 0.001; ****P < 0.0001.

The effects of different peptides on cell viability and Ang II levels under HG conditions are illustrated (Figure 5). Compared with NG group, the proliferation activity of HG group was significantly increased. After treatment with five peptides, the proliferation of VSMCs was significantly reduced in a dose-dependent manner. HLLVNK, LEHGF, HLEVR, and LLDGR at concentrations of 0.5 mg/mL significantly enhanced cell viability, indicating that these peptides have protective effects against high-glucose-induced cell damage and can promote cell proliferation. In contrast, the protective effect of LTEVR was not significant (Figures 5A–E). Figure 5F highlights the impact of peptide combinations on Ang II levels under HG conditions, demonstrating that various combinations (such as HG + HLLVNK, HG + LTEVR) significantly reduced Ang II levels. This suggests that these peptides may have potential protective effects in mitigating cell stress and inflammation induced by high glucose. Overall, these peptides not only promoted cell proliferation but also effectively reduced cell stress by lowering Ang II levels.

Compared with the general population, people with diabetes have higher rates of cardiovascular disease morbidity and mortality (12). Hyperglycemia is a major contributor to cardiovascular disease morbidity and mortality in diabetic patients (43). Hyperglycemia increases Ang II levels, which in turn enhances the proliferation and migration of VSMCs (5, 44). In this study, under high-glucose conditions, Ang II levels and markers associated with the phenotypic switch in VSMCs, such as PCNA, MMP2, Bcl2, SM22α, and BAX, are significantly altered, suggesting glucose-induced dysfunction. The ACE-inhibitory peptides HLVNK, LTEVR, LEHGF, LLDGR, and HLEVR were found to effectively reduce VSMC viability and significantly inhibit intracellular Ang II levels. These findings indicate that these ACEI peptides may counteract the adverse vascular effects of elevated glucose by downregulating Ang II within VSMCs, highlighting their therapeutic potential for managing glucose-induced vascular complications.

### 3.6 Effect of ACEI peptides on the proliferation, migration, and apoptosis in VSMCs

**Figure 6:**
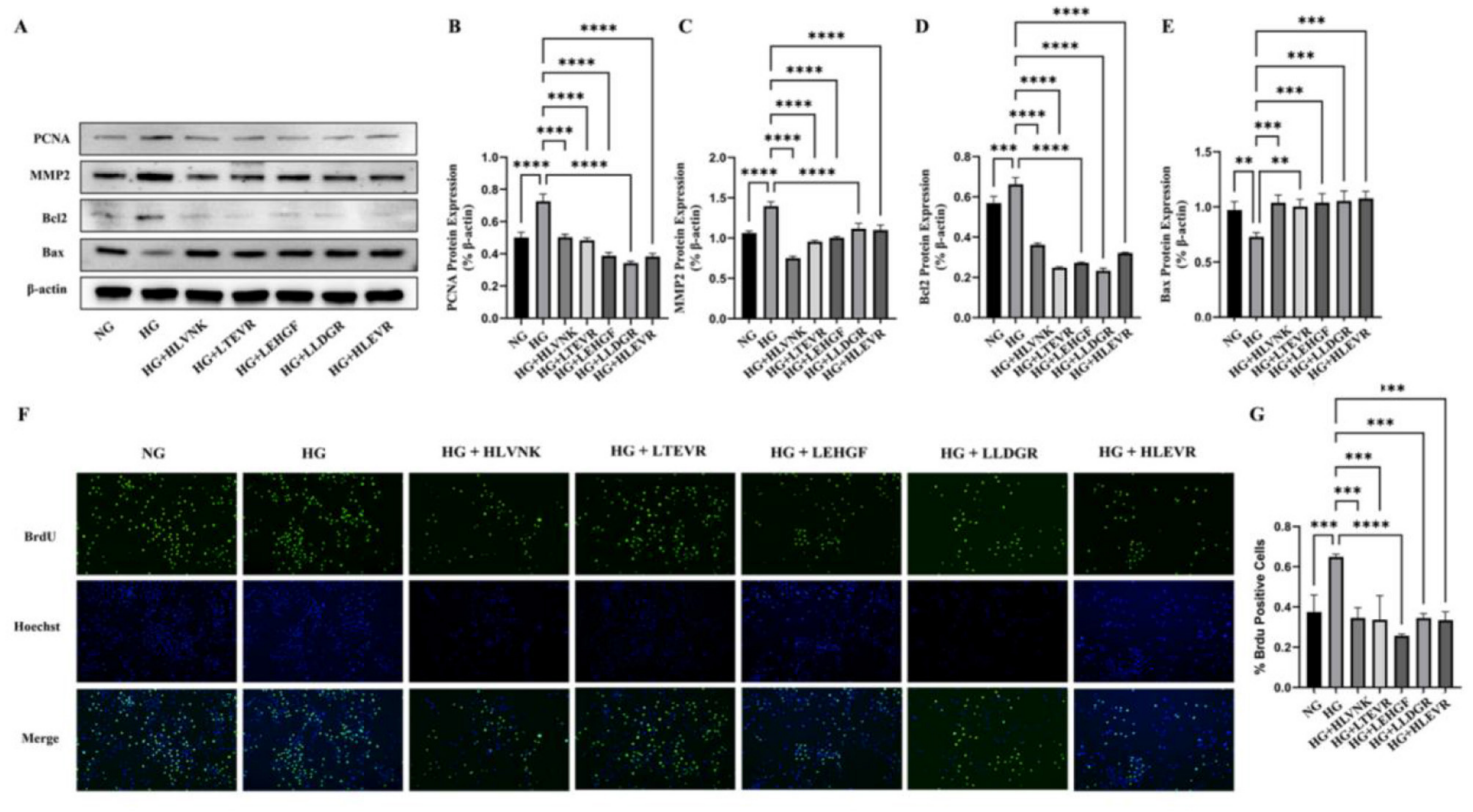
Impact of ACEI peptides on VSMC function proliferation in HG. **(A)** The effect of peptides on expression of marker of proliferation (PCNA), migration (MMP2) and apoptosis (Bcl2 and Bax) in VSMCs cultured in HG was evaluated by western blot. **(B)** Quantitative analysis of PCNA protein expression. **(C)** Quantitative analysis of MMP2 protein expression. **(D)** Quantitative analysis of Bcl2 protein expression. **(E)** Quantitative analysis of Bax protein expression. **(F)** The effect of peptides on migration of VSMCs was assessed using EdU assay. **(G)** Quantitative analysis of ACEI peptide proliferation in HG environment. *P < 0.05; **P < 0.01; ***P < 0.001; ****P < 0.0001.

The effects of the ACEI peptide on the proliferation, migration, and apoptosis of vascular smooth muscle cells are presented (Figure 6). Figures 6A–E presents the Western blot results for PCNA, MMP2, Bcl2, and Bax protein expression under different conditions. Western blot experiment showed the influence of peptides on the expression of PCNA, MMP2, Bcl2 and Bax in a HG environment (Figure 6A). In the HG group, PCNA and MMP2 expression significantly levated, respectively by 44.91 ± 3.40% (*P* < 0.0001) and 31.26 ± 1.95% (*P* < 0.0001) (Figures 6B, C), Bcl2 expression was elevated by 16.43 ± 2.03% (*P* < 0.001) (Figure 6D), and Bax expression was reduced by 24.94 ± 1.39% (*P* < 0.01) (Figure 6E). In contrast, treatment with peptides HLVNK, LTEVR, LEHGF, LLDGR, and HLEVR significantly restored the expression of these proteins, suggesting a protective role of the peptides against high glucose-induced cellular damage. Cell proliferation, which was assessed by BrdU and Hoechst staining, presented the following results in the peptide-treated groups (Figure 6F), 72.99% ± 3.61% (*P* < 0.01) increase in educational outcomes was observed in the model group when compared to the control group. Particularly HLVNK and LEHGF, showing a significant increase in BrdU-positive cells, indicating enhanced proliferation. It was confirmed that peptide treatment significantly promoted cell proliferation (Figure 6G).

**Figure 7:**
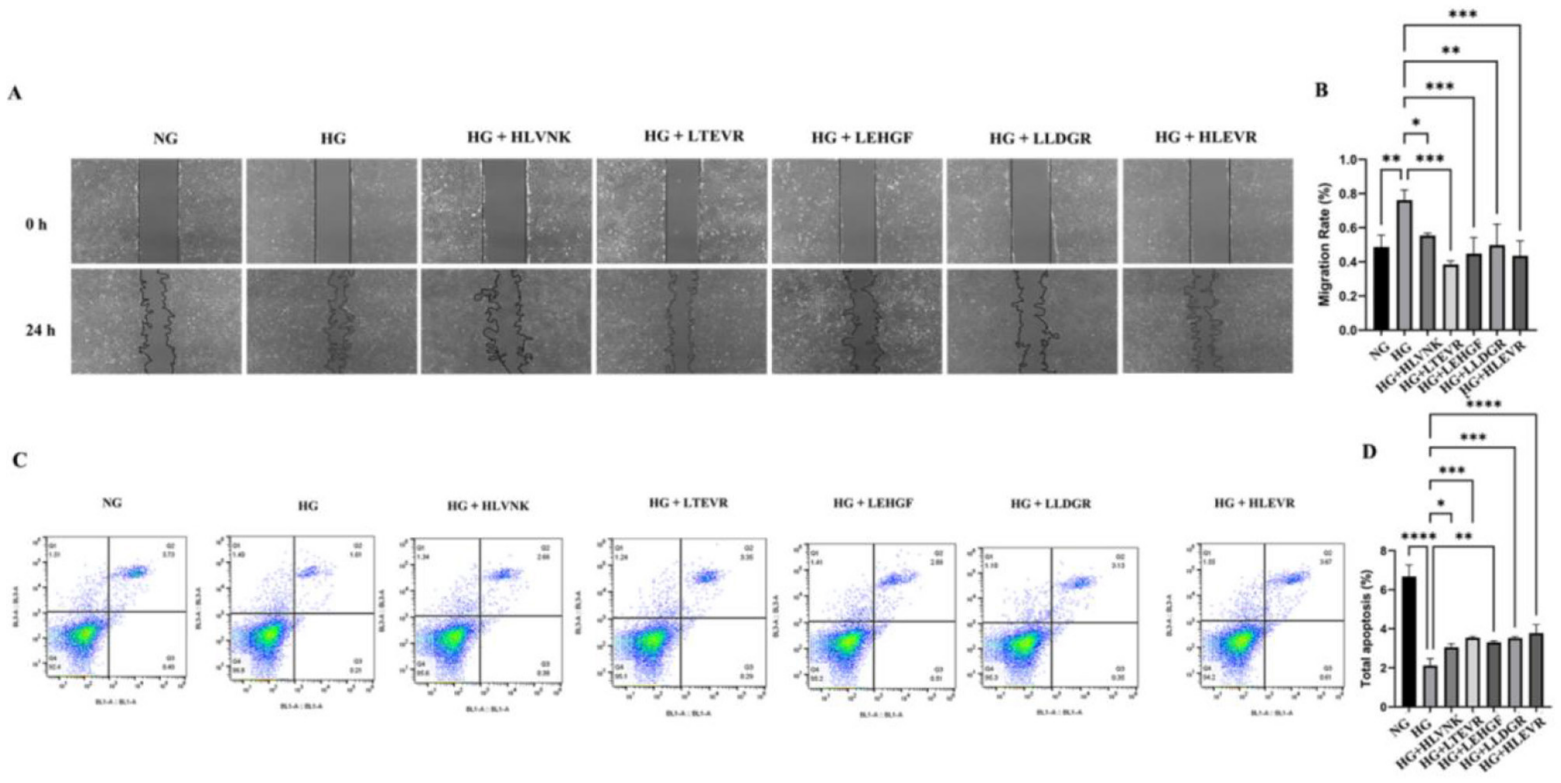
ACEI peptides influence on VSMC migration apoptosis under hg scratch assay flow cytometry. **(A)** The effect of peptides on migration of VSMCs was assessed using scratch wound assay and was shown at 0 h, 24 h. **(B)** The migration rate of peptides to VSMCs at 0 h and 24 h. **(C)** The effect of peptides on apoptosis of VSMCs was assessed using flow cytometry. **(D)** The effect of peptides on apoptosis rate of VSMCs. *P < 0.05; **P < 0.01; ***P < 0.001; ****P < 0.0001.

The results of the scratch assay indicate that peptide treatments significantly enhanced cell migration at 24 h compared to the HG group (Figure 7A). Compared with NG group, HG group increased by 56.27 ± 10.09% (*P* < 0.001). It suggested that these peptides promote cellular migration. The scratch closure rate, showing that peptide treatments significantly accelerate wound healing (Figure 7B). Finally, apoptosis was assessed via flow cytometry, revealing a significant increase in apoptosis under HG conditions, which was notably reduced by peptide treatments, confirming their protective effects in alleviating high glucose-induced apoptosis (Figures 7C, D). These peptides effectively reduced VSMC proliferation and migration, as evidenced by EdU and migration assays, and promoted apoptosis as shown through flow cytometry.

### 3.7 Effects of ACEI peptides on the activation of ERK and p38 in VSMCs

**Figure 8:**
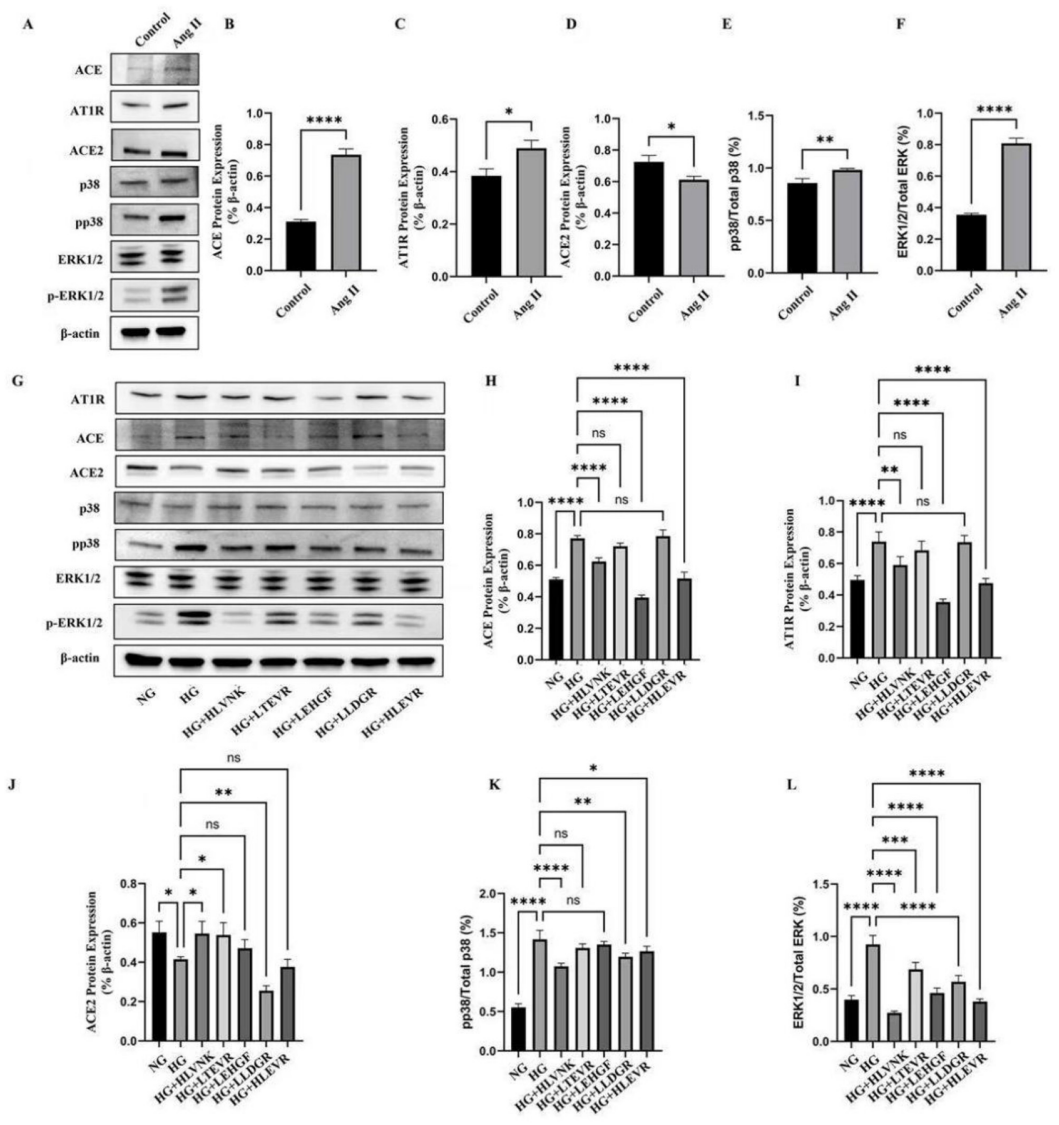
Effects of ACEI peptides on the activation of ERK and p38 under high glucose conditions. **(A)** The expression of ACE, AT1R, ACE2, p38, ERK in VSMCs induced by Ang II was evaluated by western blot. **(B)** Quantitative analysis of ACE protein expression. **(C)** Quantitative analysis of AT1R protein expression. **(D)** Quantitative analysis of ACE2 protein expression. **(E)** Quantitative analysis of pp38 protein expression. **(F)** Quantitative analysis of ERK1/2 protein expression. **(G)** The effect of peptides on expression of ACE, AT1R, ACE2, p38, ERK in VSMCs cultured in high glucose at the optimum concentration was evaluated by western blot. **(H)** Quantitative analysis of ACE protein expression. **(I)** Quantitative analysis of AT1R protein expression. **(J)** Quantitative analysis of ACE2 protein expression. **(K)** Quantitative analysis of pp38 protein expression. **(L)** Quantitative analysis of ERK1/2 protein expression. *P < 0.05; **P < 0.01; ***P < 0.001; ****P < 0.0001.

ACEI peptides modulated Ang II and ACE2 expression, enhancing cardioprotective effects by lowering blood pressure and attenuating Ang II-mediated inflammatory and proliferative signals (45–49). The results of inhibiting proliferation, migration, and inducing apoptosis indicate that these peptides have a broad regulatory effect beyond ACE inhibition, which may be achieved through the MAPK/ERK pathway, reducing phosphorylation and interfering with hyperglycemic driven smooth muscle cell and vascular remodeling signals (48, 49). The effect of ACEI peptides on ERK and p38 activation in vascular smooth muscle cells is depicted (Figure 8). The expression of AT1R, ACE, ERK1/2/Total ERK, and pp38/Total p38 was upregulated by 27.31 ± 0.020%, 136.50 ± 0.017%, 128.17 ± 0.028%, and 14.58 ± 0.041%, respectively, ACE2 expression was downregulated by 15.60 ± 0.028%. Western blot results indicated that Ang II treatment significantly altered the expression levels of ACE, AT1R, ACE2, p38, p-p38, and ERK1/2 (Figure 8A), suggesting that Ang II modulates cellular physiology through these signaling molecules and pathways. Specifically, quantitative analysis revealed that Ang II markedly upregulated ACE (Figure 8B) and AT1R (Figure 8C), implying that Ang II exerts its biological effects by enhancing the expression of these key proteins (50). Additionally, ACE2 expression was significantly reduced following Ang II treatment (Figure 8D), indicating that Ang II may exacerbate metabolic disruptions or cellular damage through negative regulation of ACE2. The activation of the p38 signaling pathway (Figure 8E), demonstrated by increased p-p38 expression, underscores the importance of this pathway in Ang II-induced stress responses (51). The ERK1/2 pathway was also affected (Figure 8F), with elevated p-ERK1/2 expression suggesting a role in promoting stress responses through Ang II-induced signaling.

Figure 8G showed variations in protein expression under high glucose and peptide treatment conditions, illustrating the regulatory effects on ACE, AT1R, ACE2, and p38. Notably, peptide treatments, especially HGLVNK and HGLTEVR, modulated ACE expression (Figure 8H), indicating that these peptides partially counteracted Ang II-induced upregulation of ACE. Changes in AT1R expression in peptide-treated groups (Figure 8I) suggested that peptides might regulate AT1R levels to mitigate the effects of Ang II. ACE2 expression was restored in peptide-treated groups (Figure 8J), indicating that peptides could oppose the negative effects of Ang II by enhancing ACE2 expression. Additionally, reduced p-p38 expression in peptide-treated groups (Figure 8K) suggested that these peptides suppressed excessive p38 pathway activation, thereby alleviating cellular stress responses. The regulation of ERK1/2 activation (Figure 8L) in peptide-treated groups, reflected by decreased p-ERK1/2 expression, further suggested.

## 4 Conclusion

In conclusion, this study discloses that the ACE inhibitory activity of broccoli protein undergoes a significant enhancement following *in vitro* digestion. Through the utilization of a combined approach of peptidomics and molecular docking analysis, five novel and highly stable peptides were screening successfully as HLVNK, LEHGF, LLDGR, HLEVR, and LTEVR, respectively. These peptides not only inhibited the proliferation, migration, and apoptosis of VSMCs by inhibiting ERK and p38 MAPK phosphorylation but also restrained the activities of ACE and AT1R, thereby prominently reducing the Ang II levels within VSMCs under high glucose. Thus, this research not only provides valuable insights into the production of novel ACEI peptides derived from broccoli protein but also offers directions for the utilization of these antihypertensive peptides in health applications.

## Data Availability

The datasets presented in this study can be found in online repositories. The names of the repository/repositories and accession number(s) can be found in the article/Supplementary material.

## References

[1] Kanugula AK Kaur J Batra J Ankireddypalli AR Velagapudi R. Renin-angiotensin system: updated understanding and role in physiological and pathophysiological states. Cureus. (2023) 15:e40725. 10.7759/cureus.40725 37350982 PMC10283427

[2] Khurana V Goswami B. Angiotensin converting enzyme (ACE). Clin Chim Acta. (2022) 524: 113-122. 10.1016/j.cca.2021.10.029 34728179

[3] Beuse A Deissler HL Hollborn M Unterlauft JD Busch C Rehak M. Different responses of the MIO-M1 Mueller cell line to angiotensin II under hyperglycemic or hypoxic conditions. Biomed Rep. (2023) 19:62. 10.3892/br.2023.1644 37614982 PMC10442740

[4] Sun P Xu N Li Y Han Y. Destruction of the blood-retina barrier in diabetic retinopathy depends on angiotensin-converting enzyme-mediated TGF-β1/Smad signaling pathway activation. Int Immunopharmacol. (2020) 85:106686. 10.1016/j.intimp.2020.106686 32531714

[5] Das S Senapati P Chen Z Reddy MA Ganguly R Lanting L. Regulation of angiotensin II actions by enhancers and super-enhancers in vascular smooth muscle cells. Nat Commun. (2017) 8:1467. 10.1038/s41467-017-01629-7 29133788 PMC5684340

[6] Wang N Xu F Lu S Zhang N Sun Y. Septin4 as an autophagy modulator regulates Angiotensin-II mediated VSMCs proliferation and migration. Biochem Biophys Res Commun. (2020) 525:272–9. 10.1016/j.bbrc.2020.02.064 32085901

[7] Jia S Ma WD Zhang CY Zhang Y Yao ZH Quan XH. Tanshinone IIA attenuates high glucose induced human VSMC proliferation and migration through miR-21-5p-mediated tropomyosin 1 downregulation. Arch Biochem Biophys. (2019) 677:108154. 10.1016/j.abb.2019.108154 31672498

[8] Zhao X Tan F Cao X Cao Z Li B Shen Z Tian Y. PKM2-dependent glycolysis promotes the proliferation and migration of vascular smooth muscle cells during atherosclerosis. Acta Biochim Biophys Sin. (2020) 52:9–17. 10.1093/abbs/gmz135 31867609

[9] Zhang W Chen S Zhang Z Wang C Liu C. FAM3B mediates high glucose-induced vascular smooth muscle cell proliferation and migration via inhibition of miR-322-5p. Sci Rep. (2017) 7:2298. 10.1038/s41598-017-02683-3 28536423 PMC5442163

[10] Ding Z Chen K Chen Y. Research on ACEI of low-molecular-weight peptides from Hirudo Nipponia Whitman. Molecules. (2022) 27:5421. 10.3390/molecules27175421 36080189 PMC9457961

[11] Nicolas-Espinosa J Carvajal M. Genome-wide identification and biological relevance of broccoli aquaporins. Plant Genome. (2022) 15:e20262. 10.1002/tpg2.20262 36263901 PMC12807285

[12] Ferreira SS Passos CP Cardoso SM Wessel DF Coimbra MA. Microwave assisted dehydration of broccoli by-products and simultaneous extraction of bioactive compounds. Food Chem. (2018) 246:386–93. 10.1016/j.foodchem.2017.11.053 29291863

[13] Chen H Xia L Zhang X. Antioxidant and hypolipidemic potential of peptides from broccoli stems and leaves. Curr Top Nutraceut Res. (2018) 18:16-20. 10.37290/ctnr2641-452X.18:16-20

[14] Li Y Pan D Zhang W Xie X Dang Y Gao X. Identification and molecular mechanism of novel ACE inhibitory peptides from broccoli protein. Food Biosci. (2024) 61:104678. 10.1016/j.fbio.2024.104678

[15] Zhou T Liu Z Pei J Pan D Gao X Dang Y. Novel broccoli-derived peptides hydrolyzed by trypsin with dual-angiotensin I-converting enzymes and dipeptidyl peptidase-IV-inhibitory activities. J Agric Food Chem. (2021) 69:10885–92. 10.1021/acs.jafc.1c02985 34494818

[16] Picariello G Siano F Di Stasio L Mamone G Addeo F Ferranti P. Structural properties of food proteins underlying stability or susceptibility to human gastrointestinal digestion. Curr Opin Food Sci. (2023) 50:100992. 10.1016/j.cofs.2023.100992

[17] Ahmed T Sun X Udenigwe CC. Role of structural properties of bioactive peptides in their stability during simulated gastrointestinal digestion: a systematic review. Trends Food Sci Technol. (2022) 120:265–73. 10.1016/j.tifs.2022.01.008

[18] Angelis ID Turco L. Caco-2 cells as a model for intestinal absorption. Curr Protoc Toxicol. (2011) 20:Unit20.26. 10.1002/0471140856.tx2006s47 21400683

[19] Fan H Xu Q Hong H Wu J. Stability and transport of spent hen-derived ACE-inhibitory peptides IWHHT, IWH, and IW in human intestinal Caco-2 cell monolayers. J Agric Food Chem. (2018) 66:11347–54. 10.1021/acs.jafc.8b03956 30280571

[20] Zhao T Liu B Yuan L Sun L Zhuang Y. ACE inhibitory activity *in vitro* and antihypertensive effect *in vivo* of LSGYGP and its transepithelial transport by Caco-2 cell monolayer. J Funct Foods. (2019) 61:103488. 10.1016/j.jff.2019.103488

[21] Touyz RM Schiffrin EL. Signal transduction mechanisms mediating the physiological and pathophysiological actions of angiotensin II in vascular smooth muscle cells. Pharmacol Rev. (2000) 52:639–72. 10.1016/S0031-6997(24)01471-6 11121512

[22] Zimmerman MC Lazartigues E Lang JA Sinnayah P Ahmad IM Spitz DR. Superoxide mediates the actions of angiotensin II in the central nervous system. Circ Res. (2002) 91:1038–5. 10.1161/01.RES.0000043501.47934.FA 12456490

[23] Lupi R Del Guerra S Bugliani M Boggi U Mosca F Torri S. The direct effects of the angiotensin-converting enzyme inhibitors, zofenoprilat and enalaprilat, on isolated human pancreatic islets. Eur J Endocrinol. (2006) 154:355–61. 10.1530/eje.1.02086 16452552

[24] Hao L Gao X Zhou T Cao J Sun Y Dang Y. Angiotensin I-converting enzyme (ACE) inhibitory and antioxidant activity of umami peptides after *in vitro* gastrointestinal digestion. J Agric Food Chem. (2020) 68:8232–41. 10.1021/acs.jafc.0c02797 32662986

[25] Dang Y Zhou T Hao L Cao J Sun Y Pan D. *In vitro* and *in vivo* studies on the angiotensin-converting enzyme inhibitory activity peptides isolated from broccoli protein hydrolysate. J Agric Food Chem. (2019) 67:6757–64. 10.1021/acs.jafc.9b01137 31184153

[26] Pei J Liu Z Pan D Zhao Y Dang Y Gao X. Transport, stability, and *in vivo* hypoglycemic effect of a broccoli-derived DPP-IV inhibitory peptide VPLVM. J Agric Food Chem. (2022) 70:4934–41. 10.1021/acs.jafc.1c08191 35436096

[27] Li Y Gao X Pan D Liu Z Xiao C Xiong Y. Identification and virtual screening of novel anti-inflammatory peptides from broccoli fermented by *Lactobacillus* strains. Front Nutr. (2022) 9:1118900. 10.3389/fnut.2022.1118900 36712498 PMC9875028

[28] Chen M Pan D Zhou T Gao X Dang Y. Novel umami peptide IPIPATKT with dual dipeptidyl peptidase-iv and angiotensin I-converting enzyme inhibitory activities. J Agric Food Chem. (2021) 69:5463–70. 10.1021/acs.jafc.0c07138 33949854

[29] Yin J Liu W Wu M Chen M Pei X He Y. Characterization of selenium-containing broccoli (*Brassica oleracea* L. var. italica planch) proteins and evaluation of antioxidant activity by electron spin resonance. Food Chem. (2024) 456:140065. 10.1016/j.foodchem.2024.140065 38878541

[30] Habinshuti I Nsengumuremyi D Muhoza B Ebenezer F Yinka Aregbe A. Recent and novel processing technologies coupled with enzymatic hydrolysis to enhance the production of antioxidant peptides from food proteins: a review. Food Chem. (2023) 423:136313. 10.1016/j.foodchem.2023.136313 37182498

[31] Martínez-Maqueda D Miralles B Recio I Hernández-Ledesma B. Antihypertensive peptides from food proteins: a review. Food Funct. (2012) 3:350–61. 10.1039/c2fo10192k 22249830

[32] Aebersold R Mann M. Mass-spectrometric exploration of proteome structure and function. Nature. (2016) 537:347–55. 10.1038/nature19949 27629641

[33] Peng J Zhang H Niu H Wu Ra. Peptidomic analyses: the progress in enrichment and identification of endogenous peptides. TrAC Trends Anal Chem. (2020) 125:115835. 10.1016/j.trac.2020.115835

[34] Xiang L Qiu Z Zhao R Zheng Z Qiao X. Advancement and prospects of production, transport, functional activity and structure-activity relationship of food-derived angiotensin converting enzyme (ACE) inhibitory peptides. Crit Rev Food Sci Nutr. (2023) 63:1437–63. 10.1080/10408398.2021.1964433 34521280

[35] Li J Hu H Chen F Yang C Yang W Pan Y. Characterization, mechanisms, structure-activity relationships, and antihypertensive effects of ACE inhibitory peptides: rapid screening from sufu hydrolysate. Food Funct. (2024) 15:9224–34. 10.1039/D4FO02834A 39158526

[36] Venkatesh R Srinivasan K Singh SA. Effect of arginine:lysine and glycine:methionine intake ratios on dyslipidemia and selected biomarkers implicated in cardiovascular disease: a study with hypercholesterolemic rats. Biomed Pharmacother. (2017) 91:408–14. 10.1016/j.biopha.2017.04.072 28475919

[37] Vallabha VS Tapal A Sukhdeo SV K G Tiku PK. Effect of arginine : lysine ratio in free amino acid and protein form on l-NAME induced hypertension in hypercholesterolemic Wistar rats. RSC Adv. (2016) 6:73388–98. 10.1039/C6RA13632J

[38] Zhang Y Cong J Bao G Gu S Wang X. *In vitro* gastrointestinal digestion study and identification of novel angiotensin I-converting enzyme inhibitory peptide from broccoli (brassica oleracea). LWT. (2022) 164:113613. 10.1016/j.lwt.2022.113613

[39] Olsen JV Ong S-E Mann M. Trypsin cleaves exclusively c-terminal to arginine and lysine residues^*^. Mol Cell Proteom. (2004) 3:608–14. 10.1074/mcp.T400003-MCP200 15034119

[40] Ningrum S Sutrisno A Hsu J-L. An exploration of angiotensin-converting enzyme (ACE) inhibitory peptides derived from gastrointestinal protease hydrolysate of milk using a modified bioassay-guided fractionation approach coupled with in silico analysis. J Dairy Sci. (2022) 105:1913–28. 10.3168/jds.2021-21112 35086704

[41] Zaky AA Simal-Gandara J Eun J-B Shim J-H Abd El-Aty AM. Bioactivities, applications, safety, and health benefits of bioactive peptides from food and by-products: a review. Front Nutr. (2022) 8:815640. 10.3389/fnut.2021.815640 35127796 PMC8810531

[42] Wang Y Fan Y Song Y Han X Fu M Wang J. Angiotensin II induces apoptosis of cardiac microvascular endothelial cells via regulating PTP1B/PI3K/Akt pathway. In Vitro Cell Dev Biol Anim. (2019) 55:801–11. 10.1007/s11626-019-00395-8 31502193

[43] Poznyak A Grechko AV Poggio P Myasoedova VA Alfieri V Orekhov AN. The diabetes mellitus-atherosclerosis connection: the role of lipid and glucose metabolism and chronic inflammation. Int J Mol Sci. (2020) 21:51835. 10.3390/ijms21051835 32155866 PMC7084712

[44] Yuan T Yang T Chen H Fu D Hu Y Wang J. New insights into oxidative stress and inflammation during diabetes mellitus-accelerated atherosclerosis. Redox Biol. (2019) 20:247–60. 10.1016/j.redox.2018.09.025 30384259 PMC6205410

[45] Yu D-C Chen X-Y Zhou H-Y Yu D-Q Yu X-L Hu Y-C. TRIP13 knockdown inhibits the proliferation, migration, invasion, and promotes apoptosis by suppressing PI3K/AKT signaling pathway in U2OS cells. Mol Biol Rep. (2022) 49:3055–64. 10.1007/s11033-022-07133-6 35032258

[46] Bertheloot D Latz E Franklin BS. Necroptosis, pyroptosis and apoptosis: an intricate game of cell death. Cell Mol Immunol. (2021) 18:1106–21. 10.1038/s41423-020-00630-3 33785842 PMC8008022

[47] Singh R Letai A Sarosiek K. Regulation of apoptosis in health and disease: the balancing act of BCL-2 family proteins. Nat Rev Mol Cell Biol. (2019) 20:175–93. 10.1038/s41580-018-0089-8 30655609 PMC7325303

[48] Lavoie H Gagnon J Therrien M. ERK signalling: a master regulator of cell behaviour, life and fate. Nat Rev Mol Cell Biol. (2020) 21:607–32. 10.1038/s41580-020-0255-7 32576977

[49] Deschênes-Simard X Malleshaiah M Ferbeyre G. Extracellular signal-regulated kinases: one pathway, multiple fates. Cancers. (2024) 16. 10.20944/preprints202311.1711.v1 38201521 PMC10778234

[50] Escobar E Rodríguez-Reyna TS Arrieta O Sotelo J. Angiotensin II, cell proliferation and angiogenesis regulator: biologic and therapeutic implications in cancer. Curr Vasc Pharmacol. (2004) 2:385–99. 10.2174/1570161043385556 15320819

[51] Corre I Paris F Huot J. The p38 pathway, a major pleiotropic cascade that transduces stress and metastatic signals in endothelial cells. Oncotarget. (2017) 8:55684–714. 10.18632/oncotarget.18264 28903453 PMC5589692

