# Supplementary material for: In vitro gastrointestinal digestion simulation screening of novel ACEI peptides from broccoli: mechanism in high glucose-induced VSMCs dysfunction
Source: Front Nutr. 2025 Jan 27;12:1528184. doi: 10.3389/fnut.2025.1528184 (PMC11807808; doi:10.3389/fnut.2025.1528184)
Supplement: Supplementary file 1 [file Table_1.docx]

**Table S1** Frequency of broccoli peptide occurrence.

**Table S2** Proteomic results of broccoli protein.

**Table S3** Mass spectrometry results of broccoli peptides.

**Table S1** Frequency of broccoli peptide occurrence.

| Sequence | Number | Length |
| --- | --- | --- |
| LLLR | 2087 | 4 |
| DLLR | 1579 | 4 |
| KLLR | 1472 | 4 |
| LELR | 1178 | 4 |
| LLPK | 1171 | 4 |
| PLLR | 1102 | 4 |
| LDLR | 1098 | 4 |
| HLLR | 656 | 4 |
| PVLK | 645 | 4 |
| LPAK | 616 | 4 |
| LHLK | 554 | 4 |
| FLVR | 534 | 4 |
| YLLR | 512 | 4 |
| PGLK | 509 | 4 |
| EPLR | 504 | 4 |
| FDLR | 430 | 4 |
| FELR | 390 | 4 |
| LEFR | 327 | 4 |
| PVGK | 303 | 4 |
| FGFK | 292 | 4 |
| HLVR | 250 | 4 |
| LDFR | 238 | 4 |
| DTPR | 227 | 4 |
| FVYR | 180 | 4 |
| LDYR | 145 | 4 |
| LAALR | 134 | 5 |
| LEDLR | 130 | 5 |
| LDLLR | 115 | 5 |
| LVLLR | 115 | 5 |
| LDVLR | 113 | 5 |
| LALAR | 107 | 5 |
| YDDR | 106 | 4 |
| APLLR | 87 | 5 |
| VDLLR | 77 | 5 |
| LGGLR | 76 | 5 |
| LTEVR | 73 | 5 |
| VPVLK | 73 | 5 |
| PAWK | 70 | 4 |
| LELPK | 69 | 5 |
| KLPLK | 66 | 5 |
| LLLPK | 65 | 5 |
| HLLPK | 63 | 5 |
| VPGLK | 61 | 5 |
| LENVR | 60 | 5 |
| LLDGR | 59 | 5 |
| LLGFK | 59 | 5 |
| VELGK | 57 | 5 |
| LKFPL | 55 | 5 |
| LPALR | 55 | 5 |
| VNLLR | 53 | 5 |
| TLLVR | 52 | 5 |
| TLKPK | 47 | 5 |
| AVLDR | 46 | 5 |
| LPVLR | 41 | 5 |
| LDFPK | 39 | 5 |
| VPVVR | 38 | 5 |
| PPVGK | 35 | 5 |
| LFKPK | 31 | 5 |
| GPPHGIQ | 31 | 7 |
| FQGPPHGIQ | 31 | 9 |
| FQGPPHGIQV | 31 | 10 |
| PTLKY | 30 | 5 |
| LFPSR | 29 | 5 |
| FDPVR | 25 | 5 |
| VLFPK | 24 | 5 |
| LVDSLK | 24 | 6 |
| DDENVNSQPF | 24 | 10 |
| VSFDPK | 23 | 6 |
| AHLDATTVL | 23 | 9 |
| LEHGF | 22 | 5 |
| IAYKPPSF | 22 | 8 |
| FAELR | 20 | 5 |
| LGFNPK | 19 | 6 |
| GPHFK | 18 | 5 |
| LGPNYL | 18 | 6 |
| ELEHGFV | 18 | 7 |
| FELEHGFV | 18 | 8 |
| LLHPR | 17 | 5 |
| LPFPR | 17 | 5 |
| PLVGK | 17 | 5 |
| GGDHVH | 17 | 6 |
| SGGDHVH | 17 | 7 |
| HVHAGTVVGK | 17 | 10 |
| AMHAVIDR | 16 | 8 |
| APLVPR | 15 | 6 |
| PVNAPK | 15 | 6 |
| AGIIDPTK | 15 | 8 |
| LQLPVNAPK | 15 | 9 |
| HMPALTE | 14 | 7 |
| PGVLR | 13 | 5 |
| LKFPLV | 13 | 6 |
| TVDTPR | 13 | 6 |
| GNAPGAVANR | 13 | 10 |
| HLEVR | 12 | 5 |
| HLVNK | 12 | 5 |
| VPLAR | 12 | 5 |
| YMEAR | 12 | 5 |
| EHGSTPGY | 12 | 8 |
| EHGSTPGYY | 12 | 9 |
| VPVMR | 11 | 5 |
| LPTTPR | 11 | 6 |
| DVIPFPR | 11 | 7 |
| AHGGVSVF | 11 | 8 |
| AAVPTIK | 10 | 7 |
| LLDASHR | 10 | 7 |
| DIGPDSIK | 10 | 8 |
| VNAGPPPPK | 10 | 9 |
| AELAPTHPIR | 10 | 10 |
| LYPGR | 9 | 5 |
| HSLPVK | 9 | 6 |
| VGLKPR | 9 | 6 |
| YEALAK | 9 | 6 |
| YKGPTL | 9 | 6 |
| PNGPSAL | 9 | 7 |
| TEVELGK | 9 | 7 |
| IGHVDSGK | 9 | 8 |
| VEWPK | 8 | 5 |
| YAEPR | 8 | 5 |
| LVPGLK | 8 | 6 |
| VNLKPK | 8 | 6 |
| VQVYPEK | 8 | 7 |
| ADLAHLPF | 8 | 8 |
| LSLLPR | 7 | 6 |
| LTHELR | 7 | 6 |
| VVVPSK | 7 | 6 |
| AVTDKPTL | 7 | 8 |
| FDYPR | 6 | 5 |
| TPEFR | 6 | 5 |
| FVRPEL | 6 | 6 |
| LGLLPK | 6 | 6 |
| QSWPVR | 6 | 6 |
| FLKPSVA | 6 | 7 |
| AGPVWNEK | 6 | 8 |
| FTENVDQR | 6 | 8 |
| IHDQGIAL | 6 | 8 |
| LLGPGLNK | 6 | 8 |
| LVGAVSSPK | 6 | 9 |
| AHTDAGGIIL | 6 | 10 |
| DLVGNNTPVF | 6 | 10 |
| IANMVPPFDK | 6 | 10 |
| YLVGAVSSPK | 6 | 10 |
| MMPGR | 5 | 5 |
| LLPALK | 5 | 6 |
| LVDPVR | 5 | 6 |
| VQVPAK | 5 | 6 |
| FTTHTDL | 5 | 7 |
| IGVNVPR | 5 | 7 |
| ALGQISER | 5 | 8 |
| ADMAPTHPIR | 5 | 10 |
| AHTDAGGLIL | 5 | 10 |
| GSAITGPIGK | 5 | 10 |
| LLHLHR | 4 | 6 |
| LVELPK | 4 | 6 |
| VVHLTK | 4 | 6 |
| AAIPTIK | 4 | 7 |
| ALNPIHK | 4 | 7 |
| GPDHFPF | 4 | 7 |
| LGPLEGK | 4 | 7 |
| ALVPIVAK | 4 | 8 |
| HVPGFVSK | 4 | 8 |
| STMGPPQR | 4 | 8 |
| EHGNTPGYY | 4 | 9 |
| AKPAVPIGGA | 4 | 10 |
| GLIKPGESVL | 4 | 10 |
| GLPDSPGVPK | 4 | 10 |
| VFFDWNDYLK | 4 | 10 |
| FKEPLK | 3 | 6 |
| PGYHPK | 3 | 6 |
| PVVPEVK | 3 | 7 |
| SGVLDPHF | 3 | 8 |
| GPNHITPVIL | 3 | 10 |
| VLKPPPYPLD | 3 | 10 |
| FLNPQK | 2 | 6 |
| FVDLLK | 2 | 6 |
| LASPGR | 2 | 6 |
| LDLPLK | 2 | 6 |
| LLSEPR | 2 | 6 |
| LPTAPR | 2 | 6 |
| LVVLGH | 2 | 6 |
| NVRPSL | 2 | 6 |
| APIPVGK | 2 | 7 |
| EGHQVTL | 2 | 7 |
| FHPGDAF | 2 | 7 |
| GGPHAAF | 2 | 7 |
| HIRPDVS | 2 | 7 |
| AIGQLSER | 2 | 8 |
| AVDPTNKL | 2 | 8 |
| FDHEVSVL | 2 | 8 |
| FYDPATQK | 2 | 8 |
| GGYYDFIK | 2 | 8 |
| SPTLPHLK | 2 | 8 |
| STVLPALK | 2 | 8 |
| VGLLPQYR | 2 | 8 |
| VLAPPNQK | 2 | 8 |
| AFDPVENIK | 2 | 9 |
| AGSTGGLLR | 2 | 9 |
| FGLSPVHPI | 2 | 9 |
| IGHLGNLNE | 2 | 9 |
| IHSDGITAL | 2 | 9 |
| DRPVPIPGSG | 2 | 10 |
| GVNLHPQTSL | 2 | 10 |
| LGYGPEDSHF | 2 | 10 |
| LGTPGK | 1 | 6 |
| LLEPLR | 1 | 6 |
| LVQLPK | 1 | 6 |
| VLLPQK | 1 | 6 |
| ASYPLK | 0 | 6 |
| CCDHLR | 0 | 6 |
| FLDPQK | 0 | 6 |
| FVELPR | 0 | 6 |
| KPLVVK | 0 | 6 |
| LATPGK | 0 | 6 |
| LFLPQK | 0 | 6 |
| LLHAPK | 0 | 6 |
| LLVHGK | 0 | 6 |
| LMPVQK | 0 | 6 |
| LTAPGK | 0 | 6 |
| LTLPQK | 0 | 6 |
| LVFPGR | 0 | 6 |
| PLGVPK | 0 | 6 |
| TLETHR | 0 | 6 |
| TLRPGL | 0 | 6 |
| VGLQHR | 0 | 6 |
| VPDFLK | 0 | 6 |
| VTLMPK | 0 | 6 |
| AALPTLK | 0 | 7 |
| AAVPTLK | 0 | 7 |
| ALNPLHK | 0 | 7 |
| APLPVGK | 0 | 7 |
| CCTLKPK | 0 | 7 |
| KDPTNKL | 0 | 7 |
| LLNNLAK | 0 | 7 |
| LLNNVAK | 0 | 7 |
| LTTVQQR | 0 | 7 |
| VALLPSK | 0 | 7 |
| VLLHAPK | 0 | 7 |
| VLLHTPK | 0 | 7 |
| VLLTDQK | 0 | 7 |
| VTLETHR | 0 | 7 |
| AGLLDPTK | 0 | 8 |
| LAYKPPSF | 0 | 8 |
| LGHVDSGK | 0 | 8 |
| LPPTHPLR | 0 | 8 |
| LQSNPLHK | 0 | 8 |
| LTTSPLHK | 0 | 8 |
| LVLDHSVQ | 0 | 8 |
| MVLPDALK | 0 | 8 |
| VSQLDSVK | 0 | 8 |
| VVLVAAHR | 0 | 8 |
| YPVLPLPK | 0 | 8 |
| AFDPVENLK | 0 | 9 |
| LDLGPDSLK | 0 | 9 |
| LLVDYLQDK | 0 | 9 |
| LLVGGELTK | 0 | 9 |
| LLVNYLQDK | 0 | 9 |
| LNLGPDSLK | 0 | 9 |
| LANMVPPFDK | 0 | 10 |
| LAYKPPSFTQ | 0 | 10 |
| LVLEGLHPMF | 0 | 10 |
| LVLLPAGVPR | 0 | 10 |
| NATAGGLLSK | 0 | 10 |
| TFQGPPHGLQ | 0 | 10 |
| VFFNWNDYLK | 0 | 10 |
| YNYLGNNPAK | 0 | 10 |

**Table S2** Proteomic results of broccoli protein.

| Entry Name | Number of matched peptides | Length | Gene Names |
| --- | --- | --- | --- |
| A0A249RRZ5_BRAOT | 77 | 479 | rbcL |
| C6KEK2_BRAOT | 42 | 247 | rbcL |
| A0A1P8NRQ6_BRAOT | 17 | 187 | - |
| A0A1S6R6T1_BRAOT | 17 | 201 | GapC1 |
| A0A1P8NRQ8_BRAOT | 15 | 187 | - |
| A0A249RRV7_BRAOT | 13 | 498 | atpB |
| H6TDM4_BRAOT | 12 | 250 | APX |
| A0A249RRX4_BRAOT | 9 | 507 | atpA |
| A0A249RS20_BRAOT | 9 | 2287 | ycf2 |
| B5A7Q8_BRAOT | 7 | 356 | GS1_1 |
| Q4TU02_BRAOT | 7 | 343 | - |
| A0A249RRV5_BRAOT | 6 | 680 | rpoC1 |
| A0A3Q9DX52_BRAOT | 6 | 684 | rpoC1 |
| U6BF50_BRAOT | 6 | 213 | - |
| A0A249RRX1_BRAOT | 5 | 750 | psaA |
| A0A249RSC6_BRAOT | 5 | 1072 | rpoB |
| A0A3S9KST2_BRAOT | 5 | 486 | psbC |
| A0A249RS39_BRAOT | 5 | 1794 | ycf1 TIC214 |
| A0A249RTC0_BRAOT | 5 | 92 | rps19 |
| A0A4P2VET1_BRAOT | 5 | 1364 | - |
| A0A249RRW1_BRAOT | 4 | 1365 | rpoC2 |
| A0A249RRW7_BRAOT | 4 | 473 | psbC |
| A0A3Q9DZR3_BRAOT | 4 | 1399 | rpoC2 |
| A0A249RS15_BRAOT | 4 | 275 | rpl2 |
| A0A249RT82_BRAOT | 4 | 236 | rps2 |
| A0A3G1GWM2_BRAOT | 4 | 499 | - |
| A0A5J6X644_BRAOT | 4 | 465 | - |
| G9B9Y8_BRAOT | 4 | 393 | CHS2 |
| J7EJA7_BRAOT | 4 | 393 | - |
| L7S2X5_BRAOT | 4 | 420 | - |
| S4SGT0_BRAOT | 4 | 2149 | GIR1 |
| A0A249RS44_BRAOT | 3 | 360 | ndhA |
| A0A343IQS8_BRAOT | 3 | 548 | - |
| A0A678XDV3_BRAOT | 3 | 548 | TGG1 |
| A0A249RRX2_BRAOT | 3 | 320 | petA |
| A0A249RT74_BRAOT | 3 | 119 | rps12 |
| A0A249RTB6_BRAOT | 3 | 217 | petB |
| A0A3Q9DZQ5_BRAOT | 3 | 118 | rps12 |
| A0A3Q9E072_BRAOT | 3 | 218 | petB |
| D4P4N9_BRAOT | 3 | 498 | ProDH |
| F8U800_BRAOT | 3 | 253 | TUB6 |
| A0A3Q9E0D4_BRAOT | 3 | 188 | orf188 |
| A0A5J6X1S2_BRAOT | 3 | 472 | - |
| A0A8G0YJ03_BRAOT | 3 | 534 | - |
| M1FZG4_BRAOT | 3 | 205 | CPI-1 |
| Q8W262_BRAOT | 3 | 369 | acs3 |
| A0A249RRW2_BRAOT | 2 | 158 | ndhJ |
| A0A249RRZ9_BRAOT | 2 | 746 | ndhF |
| A0A249RT94_BRAOT | 2 | 734 | psaB |
| C6ZH71_BRAOT | 2 | 289 | COQ5-2 COQ5 |
| A0A249RRU5_BRAOT | 2 | 524 | matK |
| A0A249RRY6_BRAOT | 2 | 100 | rps14 |
| A0A249RS13_BRAOT | 2 | 508 | psbB |
| A0A249RS22_BRAOT | 2 | 160 | rpl22 |
| F8U801_BRAOT | 2 | 202 | TUB6 |
| H6TMI5_BRAOT | 2 | 583 | - |
| H6TMI6_BRAOT | 2 | 593 | - |
| A0A0H3VD81_BRAOT | 2 | 448 | - |
| A0A1P7ZDJ1_BRAOT | 2 | 466 | - |
| A0A2P1CZZ9_BRAOT | 2 | 477 | WRKY33 |
| A0A2P1D001_BRAOT | 2 | 476 | WRKY33 |
| A0A8G0YHI6_BRAOT | 2 | 533 | - |
| A0A8G0YHW7_BRAOT | 2 | 521 | - |
| A0A8G0YI06_BRAOT | 2 | 529 | - |
| A0A8G0YI09_BRAOT | 2 | 521 | - |
| D2EDQ7_BRAOT | 2 | 190 | aps1 |
| D2EDQ8_BRAOT | 2 | 62 | aps2 |
| G0ZRI6_BRAOT | 2 | 330 | PGIP1 |
| M9UVJ7_BRAOT | 2 | 499 | - |
| M9UZ88_BRAOT | 2 | 326 | - |
| Q05GM4_BRAOT | 2 | 366 | 1co-GD |
| Q8GUP7_BRAOT | 2 | 283 | lh |
| A0A249RRZ6_BRAOT | 1 | 327 | rpoA |
| A0A249RS16_BRAOT | 1 | 81 | psaC |
| A0A249RSY8_BRAOT | 1 | 225 | ndhK |
| A0A249RT21_BRAOT | 1 | 512 | ndhB |
| A0A249RTA4_BRAOT | 1 | 489 | accD |
| A0A3S9KSH9_BRAOT | 1 | 276 | ndhK |
| A0A411PAP1_BRAOT | 1 | 426 | HDA9 |
| A0A249RRX5_BRAOT | 1 | 184 | ycf4 |
| A0A249RRY1_BRAOT | 1 | 117 | rpl20 |
| A0A249RRY5_BRAOT | 1 | 66 | rpl33 |
| A0A249RRY9_BRAOT | 1 | 218 | rps3 |
| A0A249RS04_BRAOT | 1 | 160 | petD |
| A0A249RS35_BRAOT | 1 | 155 | rps7 |
| A0A249RSE8_BRAOT | 1 | 101 | rps18 |
| A0A249RT03_BRAOT | 1 | 205 | clpP |
| A0A3Q9DZM4_BRAOT | 1 | 174 | petD |
| B1Q390_BRAOT | 1 | 399 | BoA6 |
| B1Q3A4_BRAOT | 1 | 524 | BoCKX1 |
| B1Q3A5_BRAOT | 1 | 569 | BoGAI |
| K9K869_BRAOT | 1 | 287 | - |
| A0A0C4MDF4_BRAOT | 1 | 502 | - |
| A0A1B1SNP8_BRAOT | 1 | 502 | CYP83A1-1 |
| A0A1P7ZDI8_BRAOT | 1 | 501 | - |
| A0A1U9X717_BRAOT | 1 | 228 | - |
| A0A1X9RIK9_BRAOT | 1 | 328 | - |
| B1Q391_BRAOT | 1 | 49 | BoMS2A |
| B1Q392_BRAOT | 1 | 49 | BoMS2B |
| B1Q3A1_BRAOT | 1 | 332 | BoCysP1 |
| B1Q3A2_BRAOT | 1 | 441 | BoCP3 |
| B2M1R8_BRAOT | 1 | 138 | - |
| D2EDR0_BRAOT | 1 | 348 | myb28 |
| F1BXA3_BRAOT | 1 | 130 | - |
| F2X5J8_BRAOT | 1 | 280 | - |
| F2X5J9_BRAOT | 1 | 280 | - |
| F2X5K0_BRAOT | 1 | 280 | - |
| F2X5K1_BRAOT | 1 | 280 | - |
| I1UYF9_BRAOT | 1 | 632 | EMF2_1 |
| I1UYG1_BRAOT | 1 | 425 | VRN2 |
| K4EJR8_BRAOT | 1 | 285 | WRKY3 |
| L7S1M0_BRAOT | 1 | 199 | - |
| L7S6B7_BRAOT | 1 | 540 | - |
| L8B0B1_BRAOT | 1 | 190 | BO-VTC1 |
| Q05GM5_BRAOT | 1 | 335 | 1col1-GD |
| Q1ELV7_BRAOT | 1 | 135 | flc3 |
| V9Z5P8_BRAOT | 1 | 297 | FAE1 |

**Note:** - **represents unannotated genes.**

**Table S3** Mass spectrometry results of broccoli peptides

| Peptide | ALC (%) | m/z | RT | CV | Area | Mass |
| --- | --- | --- | --- | --- | --- | --- |
| HLVNK | 98.5 | 305.68573 | 13.1041 | -45 | 7.28E+07 | 609.3598 |
| HLEVR | 98.7 | 327.18869 | 16.6696 | -45 | 4.12E+07 | 652.3657 |
| LLDGR | 96.3 | 287.17026 | 19.7281 | -45 | 2.54E+07 | 572.3282 |
| LTEVR | 96.1 | 309.18326 | 16.7391 | -45 | 2.49E+07 | 616.3544 |
| LEHGF | 95 | 602.29175 | 24.3494 | -45 | 1.13E+07 | 601.286 |
